# Reduction in Acetylation of Superoxide Dismutase 2 in Skeletal Muscle Improves Exercise Capacity in Mice With Heart Failure

**DOI:** 10.1002/jcsm.13850

**Published:** 2025-06-13

**Authors:** Tomoka Masunaga, Tomoyasu Suenaga, Shouji Matsushima, Toru Hashimoto, Shingo Takada, Eri Noda, Yoshizuki Fumoto, Soichiro Hata, Takashi Yokota, Shintaro Kinugawa

**Affiliations:** ^1^ Department of Cardiovascular Medicine, Faculty of Medical Sciences Kyushu University Fukuoka Japan; ^2^ Division of Cardiovascular Medicine, Research Institute of Angiocardiology, Faculty of Medical Sciences Kyushu University Fukuoka Japan; ^3^ Department of Lifelong Sport, School of Sports Education Hokusho University Ebetsu Japan; ^4^ Department of Molecular Biology Hokkaido University Graduate School of Medicine Sapporo Japan; ^5^ Institute of Health Science Innovation for Medical Care Hokkaido University Hospital Sapporo Japan; ^6^ Department of Cardiovascular Medicine NHO Fukuoka National Hospital Fukuoka Japan

**Keywords:** acetylation, exercise intolerance, heart failure, skeletal muscle, superoxide dismutase

## Abstract

**Background:**

Skeletal muscle abnormalities, including mitochondrial dysfunction, play a crucial role in decreasing exercise capacity in patients with heart failure (HF). Although enhanced reactive oxygen species (ROS) production in skeletal muscle mitochondria has been implicated in skeletal muscle abnormalities, the underlying mechanisms have not been fully elucidated to date. Superoxide dismutase 2 (SOD2), an antioxidant enzyme present in mitochondria, is modified by acetylation, which reduces its activity. The aim of this study was to clarify whether reducing SOD2 acetylation by sirtuins 3 (SIRT3) activation improves skeletal muscle mitochondrial function and exercise capacity in HF model mice.

**Methods:**

Myocardial infarction (MI) by ligation of the coronary artery or sham surgery was performed in male C57BL/6 J mice. Two weeks after surgery, these mice were treated with either the SIRT3 activator Honokiol (5 mg/kg body weight/day, i.p.) or vehicle. After 2 weeks of treatment, exercise capacity was evaluated by the treadmill test. Gastrocnemius muscle samples collected from the mice were used to measure mitochondrial function, as well as the levels of SIRT3, acetylated SOD2, and ROS production. Finally, the effect of adeno‐associated virus serotype 9 (AAV9)‐mediated overexpression of SIRT3 in the skeletal muscle on the exercise capacity of MI mice was investigated.

**Results:**

MI mice showed decreased cardiac function and skeletal muscle weight, but Honokiol did not affect these. Exercise capacity was significantly decreased in MI mice compared with sham mice by 24.9%, and Honokiol treatment improved the exercise capacity of MI mice by 40.4% (*p* < 0.05). The mitochondrial oxygen consumption rate was impaired in MI mice, but was improved by Honokiol treatment. SIRT3 expression was decreased by 26.8%, and SOD2 acetylation was increased by 36.9% in the skeletal muscle of MI mice compared with sham (*p* < 0.05), and Honokiol treatment resulted in complete recovery of these levels (*p* < 0.05). Consistent with SOD2 acetylation, ROS production in the skeletal muscle was increased in MI mice and was ameliorated by Honokiol (*p* < 0.05). SIRT3 expression was increased in MI + AAV9‐SIRT3 mice compared with MI + AAV9‐Control mice. The overexpression of SIRT3 improved exercise capacity without altering cardiac function.

**Conclusions:**

The SIRT3 activator Honokiol improved exercise capacity in MI model mice with HF, by improving mitochondrial function in skeletal muscle through the reduction of SOD2 acetylation. SIRT3 activation may thus be a novel therapeutic target for improving exercise capacity in patients with HF.

## 1 Introduction

Decreased exercise tolerance is a hallmark of patients with heart failure (HF), and is an independent predictor of an unfavorable prognosis [1, 2]. Peak oxygen uptake, which is an indicator of exercise tolerance, is determined by various factors. Among these, skeletal muscle abnormalities are the most important determining factor for the decrease in peak oxygen uptake in HF patients [1]. It is known that skeletal muscle abnormalities, such as a shift in skeletal muscle fiber type, impaired energy metabolism, mitochondrial dysfunction, skeletal muscle atrophy, and muscle weakness occur in patients with HF [1, 3, 4, 5]. However, to date there is no effective treatment for skeletal muscle abnormalities other than exercise therapy.

Although much remains unknown regarding the molecular mechanisms underlying skeletal muscle abnormalities in HF, an increase in reactive oxygen species (ROS) is thought to be involved [3, 6]. Skeletal muscle biopsies from HF patients showed increased ROS production, which was associated with abnormal mitochondrial function and morphology [7]. This has also been shown in a mouse HF model after myocardial infarction (MI) [8, 9]. The main sources of ROS in skeletal muscle are nicotinamide adenine dinucleotide phosphate (NADPH) oxidase 2 [10], xanthine oxidase [9] and mitochondria, and ROS directly cause mitochondrial damage, leading to a further increase in mitochondria‐derived ROS. ROS were also associated with a decrease in slow twitch fibre [9]. Furthermore, superoxide dismutase 2 (SOD2), an antioxidant enzyme present within mitochondria, also plays an important role in skeletal muscle. We found that SOD2 heterozygous knockout mice have increased ROS production in skeletal muscle and impaired exercise capacity [11].

Acetylation is a major posttranslational modification of proteins and regulates protein function [12]. In particular, it has been reported that more than 65% of mitochondrial proteins undergo acetylation [13]. Acetylation modifications are reversibly regulated by acetyltransferases and nicotinamide adenine dinucleotide oxidized form (NAD^+^)‐dependent deacetylases (sirtuins). There are seven sirtuin subtypes, and among these, sirtuin 3 (SIRT3) is the major deacetylase in mitochondria [14]. The activity of SOD2 is regulated by the acetylation of lysine residues [15], and the lack of SIRT3 reduces SOD2 activity through the acetylation of SOD2 [16]. We previously showed that SIRT3 expression in skeletal muscle is decreased, and the acetylation of skeletal muscle mitochondrial proteins is increased in a mouse model of HF after MI [17], and this may be associated with impaired mitochondrial function in the skeletal muscle.

In this study, we used Honokiol, a natural biphenolic compound derived from the bark of magnolia trees, as a pharmacological activator of SIRT3 [18]. Honokiol treatment was shown to suppress the cardiac hypertrophy caused by pressure overload or agonists such as isoproterenol, but these effects were diminished by SIRT3 knockout [19]. The effect of Honokiol in this previous study was shown to be a result of the suppression of ROS production through an increase in SIRT3 expression and a reduction in SOD2 acetylation [19]. The SIRT3‐dependent effects of Honokiol have also been reported in the acute renal impairment mouse model induced by cisplatin [20]. We hypothesised that SIRT3 activator treatment of HF model mice after MI would improve mitochondrial function and exercise capacity by reducing acetylation of the SOD2 protein.

## 2 Methods

### 2.1 Cell Culture and Transfection With Small Interfering RNA (siRNA) Against SIRT3

**FIGURE 1:**
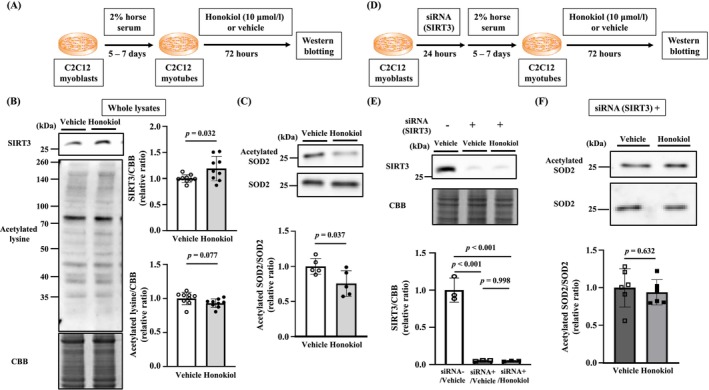
Honokiol decreases the acetylation of SOD2 in C2C12 myotubes in a SIRT3‐dependent manner. (A) Experimental protocol for Honokiol treatment of C2C12 myotubes. (B) Representative western blots (left) and summary data (right) of SIRT3 and acetylated lysine levels in whole lysates from C2C12 myotubes treated with vehicle (*n* = 9) or Honokiol (*n* = 9). Results were normalized to non‐specific bands of CBB‐stained gel. (C) Representative western blots (top) and summary data (bottom) of acetylated SOD2 and total SOD2 levels in C2C12 myotubes treated with vehicle (*n* = 5) or Honokiol (*n* = 5). Acetylated SOD2 was normalized to total SOD2. (D) Experimental protocol for Honokiol treatment of C2C12 myotubes transfected with SIRT3 siRNA. (E) Representative western blot (top) and summary data (bottom) of SIRT3 in C2C12 myotubes transfected with SIRT3 siRNA or scramble. (F) Representative western blots (top) and summary data (bottom) of acetylated SOD2 and SOD2 levels in C2C12 myotubes treated with vehicle (*n* = 6) or Honokiol (*n* = 6) with transfection of SIRT3 siRNA. Acetylated SOD2 was normalized to total SOD2. Data are shown as the mean ± SD *p* values were calculated by the unpaired Student *t*‐test or by one‐way ANOVA followed by the Tukey *post hoc* test. SIRT3, sirtuin 3; CBB, Coomassie Brilliant Blue; SOD2, superoxide dismutase 2; siRNA, small interfering RNA.

Cell culture studies were performed using mouse C2C12 myoblast cell lines purchased from the American Type Culture Collection (Manassas, VA) as previously described [17]. Their protocols were shown in Figure 1A,D. The cells were maintained in Dulbecco’s Modified Eagle's Medium (Sigma‐Aldrich, St. Louis, Missouri) containing 10% fetal bovine serum (Thermo Fisher Scientific, Waltham, Massachusetts) and 1% penicillin/streptomycin (Thermo Fisher Scientific) at 37 °C with 5% CO_2_ in air. After reaching full confluency, myoblasts were differentiated into myotubes in medium with 2% horse serum (Thermo Fisher Scientific) for 5–6days. The myotubes were treated with Honokiol (10 μmol/L, Sigma‐Aldrich) or vehicle for 72 h. Dimethyl sulfoxide (Sigma‐Aldrich) was used as a vehicle at a final volume concentration of 0.1%.

For transfection, C2C12 myoblasts were transfected with siRNA against SIRT3 (Thermo Fisher Scientific) using Lipofectamine RNAiMAX (Thermo Fisher Scientific) and incubated for 24 h. Then, the transfected myoblasts were differentiated into myotubes and treated with Honokiol or vehicle, as described above.

### 2.2 Experimental Animals

Male C57BL/6 J mice were purchased from CLEA Japan (Tokyo). Animals were used for experiments at 9–11 weeks of age (weight: 22–28 g). Mice were bred in a pathogen‐free environment, provided a standard chow diet and water, and kept under a constant 12‐h light–dark cycle at a temperature of 23°C–25°C. As it is known that there are clear sex differences in the skeletal muscle phenotypes including weight, fibre type, and capillarity, only male mice were used in the present experiments [21]. MI was induced by ligating the left coronary artery, as described previously [9, 17]. A sham operation without ligation of the coronary artery was also performed. Mice were anaesthetised with an i.p. injection of a mixture of 0.3 mg/kg of medetomidine (Kyoritsuseiyaku, Tokyo), 4.0 mg/kg of midazolam (Sandoz, Tokyo), and 5.0 mg/kg of butorphanol (Meiji Seika Kaisha, Tokyo), and the adequacy of the anaesthesia was monitored based on the disappearance of the pedal withdrawal reflex. All procedures involving animals and animal care protocols were approved by the Committee on Ethics of Animal Experiments of Kyushu University Graduate School of Medicine and Pharmaceutical Sciences (study Approval No.: A24‐129‐1) and were performed in accordance with the Guidelines for Animal Experiments of Kyushu University and the Guidelines for the Care and Use of Laboratory Animals published by the US National Institutes of Health (revised in 2011).

### 2.3 Study Protocol

**FIGURE 2:**
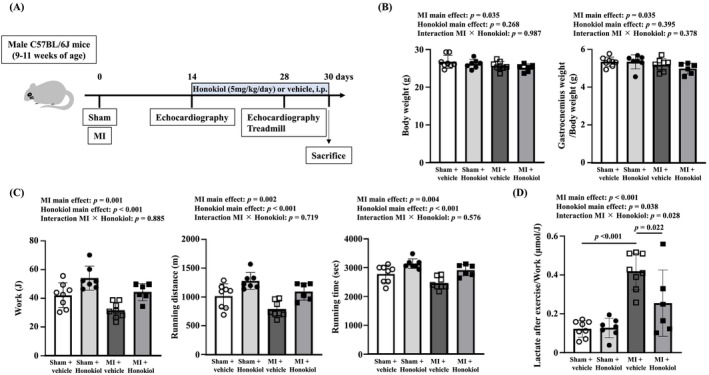
Honokiol ameliorates the reduced exercise capacity of MI mice. (A) Experimental protocol for Honokiol treatment to MI mice. Summary data of body weight and gastrocnemius weight/body weight (B), work, running distance and running time (C), and lactate production after exercise/work (D) in sham + vehicle (*n* = 8), sham + Honokiol (*n* = 7), MI + vehicle (*n* = 8) and MI + Honokiol mice (*n* = 6). Data are shown as the mean ± SD *p* values of the main effect for each factor and interaction effect between two factors were calculated by two‐way ANOVA, with the factors of MI and Honokiol, and if there was an interaction effect between 2 factors, the Tukey post hoc test was performed. MI, myocardial infarction.

Experimental protocol for Honokiol treatment to MI mice was shown in Figure 2A. Based on our past experience, we estimated the mortality rate at 4 weeks after MI to be 45%–50%, and assumed that Honokiol treatment would not affect survival rates. To ensure that the final sample size for the main analysis of exercise capacity was approximately 8, either surgery of sham or MI was randomly performed on 8, 8, 14, 14 mice in the sham + vehicle, sham + Honokiol, MI + vehicle, and MI + Honokiol group, respectively. No mice died in the sham + vehicle group. One mouse in the sham + Honokiol group lost weight for an unknown reason and was euthanized. Six mice died in the MI + vehicle group over the course of the 4‐week experiment. Two mice died within 24 h after surgery in the MI + Honokiol group, and six died over the course of the 4‐week experiment. The final analysis of exercise capacity was performed on 8, 7, 8, and 6 mice, respectively. Two weeks after the surgery, echocardiography was performed on all mice, and then, Honokiol or vehicle was administered by daily i.p. injections for an additional 2 weeks. Referring to previous reports, Honokiol was administered at the dose of 5 mg/kg body weight [22]. Four weeks after the surgery (at the end of the two‐week treatment), the four groups of mice were subjected to a treadmill test and echocardiography. Next, under deep anaesthesia induced by the above‐described drug combination, the mice were euthanized, and their hindlimb skeletal muscles were excised and weighed. In the present study, we used the gastrocnemius muscle, which contains a good balance of fast and slow twitch fibres, for analysis. To avoid direct effects of the treadmill test on the muscle analysis results, tissue sampling was performed over 2 days after the treadmill test [17]. Measurement of oxygen consumption rate, fibre type staining, measurements of citrate synthase and aconitase activities and hydrogen peroxide (H_2_O_2_) release, transmission electron microscopy analysis, Western blotting and quantitative real‐time polymerase chain reaction were performed using gastrocnemius muscle extracted from the three groups other than the sham + Honokiol group. There was a limit to the amount of gastrocnemius muscle sample that could be obtained from one mouse, and those from mice used in the main analysis were not sufficient to perform all experiments. Therefore, additional groups of mice were added as necessary to obtain gastrocnemius muscle samples. Data acquisition and analysis were performed by investigators who were blinded to the group assignment. An expanded methods section is provided in the Supporting Information section.

### 2.4 Adeno‐Associated Virus Serotype 9 (AAV9)‐Mediated Overexpression of SIRT3 in Vivo

AAV9 vectors expressing mouse SIRT3 tagged with enhanced green fluorescent protein (GFP) under the control of the human actin alpha 1 (ACTA1) promoter (AAV9‐SIRT3) or empty control (AAV9‐Control) were designed and generated (VectorBuilder, Chicago, IL). The ACTA1 promoter was chosen to ensure skeletal muscle specificity of gene transfer. The vectors (2.5 × 10^12^ viral genomes in phosphate‐buffered saline) were injected into the tail vein of mice 2 weeks after MI surgery. Four weeks after surgery 2 weeks after the treatment), MI + AAV9‐Control mice (*n* = 7) and MI + AAV9‐SIRT3 mice (*n* = 7) were subjected to the treadmill test and euthanized as described above.

### 2.5 Statistical Analysis

Data are expressed as the mean ± SD. No statistical method was used to predetermine the sample size. The number of animals used in each experiment was determined based on sample sizes commonly used in the field. The normality of the data was evaluated using the Shapiro–Wilk test. For two‐group comparisons, the statistical evaluation of normally distributed variables was performed using the unpaired Student *t*‐test, and the analysis of nondistributed variables was performed using the Mann–Whitney *U* test. For three‐group comparisons, the statistical evaluation of normally distributed variables was performed using one‐way ANOVA, followed by the Tukey test. If the data was nonnormally distributed, analysis was performed using the Kruskal‐Wallis test followed by Dunn’s multiple comparisons test. For comparisons of four groups with two factors (animal model; myocardial infarction × treatment; Honokiol), statistical analyses were performed using two‐way ANOVA, and when there was an interaction effect between two factors, the Tukey test was subsequently performed. Analyses were performed using GraphPad Prism 10 software (GraphPad, San Diego, California). A *p* value of less than 0.05 was considered to indicate a statistically significant difference between two groups.

## 3 Results

### 3.1 Changes in Mitochondrial Sirtuins Family in Skeletal Muscle After MI

The sirtuins that exist and act in mitochondria are SIRT3, SIRT4 and SIRT5 [23]. We found that there were no differences in the protein expression levels of SIRT4 and SIRT5 in the gastrocnemius muscle between sham + vehicle mice and MI + vehicle mice (Figure S1). We previously demonstrated that SIRT3 protein was decreased in the gastrocnemius muscle after MI [17]. Therefore, we focused on SIRT3 in this study.

### 3.2 Honokiol Decreased the Acetylation of SOD2 via Increasing SIRT3 Expression in C2C12 Myotubes

To clarify whether the effects of Honokiol are mediated through SIRT3, experiments in cultured C2C12 myotubes were designed. SIRT3 expression was significantly increased by the addition of Honokiol to C2C12 myotubes (Figure 1B). However, there was no significant change in the acetylation of whole protein extracted and mitochondria isolated from C2C12 myotubes treated with between vehicle and Honokiol (Figure 1B and Figure S2A). We focused on SOD2, which is within mitochondria. Honokiol did not affect SOD2 expression but attenuated the acetylation of SOD2 (Figure 1C). To further confirm that the effect of Honokiol is mediated by increasing SIRT3 expression, the effect of Honokiol was verified using C2C12 myotubes in which SIRT3 was knocked down (Figure 1E). There was no significant change in the acetylation of mitochondria isolated from C2C12 myotubes with transfection of siSIRT3 treated with between vehicle and Honokiol (Figure S2B). As expected, Honokiol did not affect the acetylation of SOD2 in these C2C12 myotubes (Figure 1F).

### 3.3 Honokiol Treatment Improved the Exercise Capacity of MI Mice

Cardiac function was evaluated by echocardiography 2 weeks after the surgery before the start of treatment. There were no differences in left ventricular diameter and fractional shortening between MI + vehicle and MI + Honokiol mice (Figure S3), indicating that there was no difference in the severity of MI before Honokiol treatment between the two groups. Four weeks after the surgery, the four groups of mice were evaluated. Body weight and gastrocnemius muscle weight were significantly decreased in MI mice compared with sham mice (Figure 2B). MI mice showed left ventricular dilation, hypertrophy, and dysfunction with an increase in lung weight/body weight (Figures S4 and S5). These data suggested that MI mice had cardiac dysfunction with lung congestion and skeletal muscle atrophy 4 weeks after the surgery. The treatment of mice with Honokiol did not affect these parameters (Figures S4 and S5). All indices of exercise capacity, which were measured by the treadmill test, including work, running distance, and running time, were significantly decreased in MI mice compared with sham mice, and Honokiol treatment improved the exercise capacity of MI mice (Figure 2C). Furthermore, lactate production immediately after exercise adjusted by work was significantly increased in MI + vehicle mice compared with sham + vehicle mice (Figure 2D), which suggests that aerobic capacity is impaired in MI mice. Honokiol reduced it only in MI mice (Figure 2D), suggesting that the effects of Honokiol on aerobic capacity are mainly exerted in MI mice. Therefore, the subsequent analyses were performed excluding the sham + Honokiol mice.

### 3.4 Honokiol Treatment Improved Impaired Mitochondrial Respiration and Decreased Fibre‐Type I in MI Mice

**FIGURE 3:**
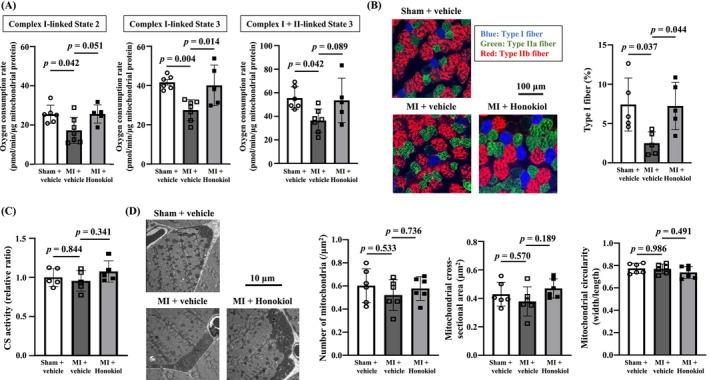
Honokiol treatment improved mitochondrial respiration and the decrease in fibre Type I in the skeletal muscle of MI mice. (A) Summary data of complex I‐linked State 2, complex I‐linked State 3, and complex I + II‐linked State 3 respiration in isolated mitochondria from the gastrocnemius tissues form sham + vehicle (*n* = 6), MI + vehicle (*n* = 7) and MI + Honokiol (*n* = 6). (B) Representative high‐power photomicrographs of gastrocnemius muscle tissue sections stained with MHC immunofluorescence staining (left, blue: Type I fibre, green: Type IIa fibre, red: Type IIb fibre), and summary data (right) of the proportion of Type I fibre to the total fibres in sham + vehicle (*n* = 5), MI + vehicle (*n* = 5), and MI + Honokiol mice (*n* = 5). (C) Summary data of citrate synthase activity in the skeletal muscle of sham + vehicle (*n* = 5), MI + vehicle (*n* = 5), and MI + Honokiol mice (*n* = 5). (D) Representative transmission electron microscopy images of gastrocnemius muscle (left) and summary data of number of mitochondria, mitochondrial cross‐sectional area and mitochondrial circularity (right) in sham + vehicle (*n* = 3), MI + vehicle (*n* = 3), and MI + Honokiol mice (*n* = 3). Six images from 3 mice per group were analysed. Data are shown as the mean ± SD *p* values were calculated by one‐way ANOVA followed by the Tukey post hoc test. MI, myocardial infarction.

Honokiol treatment of MI mice improved exercise capacity without altering their cardiac function, lung congestion or skeletal muscle volume. As Honokiol treatment was effective in improving aerobic metabolism, we hypothesised that it might improve skeletal muscle abnormalities, particularly mitochondrial function and fibre type switch. We hence assessed oxygen consumption rate in isolated mitochondria from gastrocnemius muscle. Complex I‐linked State 2 and State 3 respirations were decreased in MI + vehicle mice compared with sham + vehicle mice. Complex I‐ and II‐linked State 3 respiration was also decreased in MI + vehicle mice compared with sham + vehicle mice (Figure 3A). Complex I‐linked State 3 respiration was significantly restored by Honokiol treatment, and Complex I‐linked State 2 and Complex I‐ and II‐linked State 3 respirations tended to be restored by Honokiol treatment (Figure 3A). Next, we analysed the proportion of fibre type in the gastrocnemius muscle by the myosin heavy chain (MHC) immunofluorescence staining. The proportion of fibre Type I was significantly decreased in MI + vehicle compared with sham + vehicle mice, and Honokiol recovered it (Figure 3B). On the other hand, the proportions of fibre Type IIa and Type IIb did not change among the three groups (Figure S6A). These results were consistent with the results of MHC gene expression. Gene expression of *Myh7*, which encodes fibre type I, was significantly decreased in MI + vehicle mice compared with sham + vehicle mice and was significantly increased in MI + Honokiol mice (Figure S6B). There were no statistically significant changes in other MHC isoforms including *Myh1*, *Myh2* and *Myh4* among the three groups (Figure S6B).

In contrast, mitochondrial volume and morphology, assessed by citrate synthase activity and transmission electron microscopy, were not altered among the three groups (Figure 3C,D). Consistent with these results, there were no significant changes in the expression of proteins associated with mitochondrial biogenesis (peroxisome proliferator–activated receptor γ coactivator‐1 α (PGC‐1α), SIRT1), fusion (mitofusin1, mitofusin2 and optic atrophy1), fission (dynamin‐related protein 1 (DRP1)) and mitophagy (PTEN‐induced serine/threonine kinase 1 (PINK1), Parkin) [24] among the three groups of mice (Figure S7).

### 3.5 Honokiol Treatment Attenuated SOD2 Acetylation and ROS Production in Skeletal Muscle of MI Mice

**FIGURE 4:**
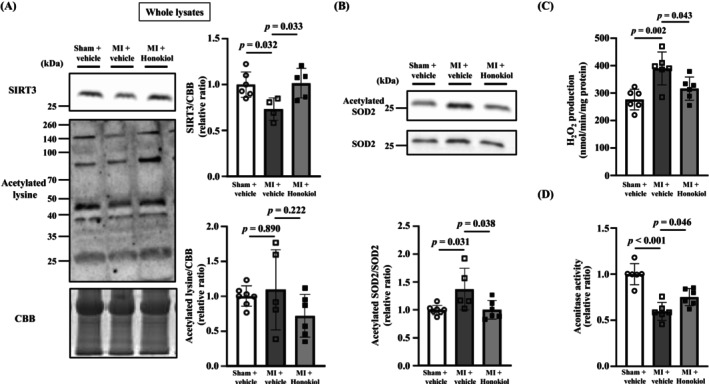
Honokiol treatment attenuated the acetylation of SOD2 and ROS production in the skeletal muscle of MI mice. (A) Representative western blots (left) and summary data (right) of SIRT3 and acetylated lysine levels in whole lysates from the skeletal muscle of sham + vehicle (*n* = 6), MI + vehicle (*n* = 4), and MI + Honokiol mice (*n* = 5). Results were normalized to non‐specific bands of the CBB‐stained gel. (B) Representative western blots (top) and summary data (bottom) of acetylated SOD2 and total SOD2 levels in the skeletal muscle of sham + vehicle (*n* = 7), MI + vehicle (*n* = 5) and MI + Honokiol mice (*n* = 6). Acetylated SOD2 was normalized to total SOD2. (C) Summary data of H_2_O_2_ production from isolated mitochondria in gastrocnemius muscle of sham + vehicle (*n* = 6), MI + vehicle (*n* = 6) and MI + Honokiol mice (*n* = 6). (D) Summary data of aconitase activity in gastrocnemius muscle of sham + vehicle (*n* = 6), MI + vehicle (*n* = 6), and MI + Honokiol mice (*n* = 6). Data are shown as the mean ± SD *p* values were calculated by one‐way ANOVA followed by the Tukey post hoc test. MI, myocardial infarction; SIRT3, sirtuin 3; CBB, Coomassie Brilliant Blue; SOD2, superoxide dismutase 2.

SIRT3 protein expression level was significantly decreased in the skeletal muscle of MI + vehicle mice compared with sham + vehicle mice and was increased in MI + Honokiol mice (Figure 4A). Despite changes in SIRT3, there was no significant change in the acetylation of whole protein extracted from the skeletal muscle among the three groups (Figure 4A). In contrast, the acetylation of mitochondrial protein extracted from the skeletal muscle was increased in MI + vehicle mice compared with sham + vehicle mice (Figure S8). However, Honokiol did not decrease the acetylation of mitochondrial proteins in MI mice (Figure S8). The acetylation of SOD2 was significantly increased in the skeletal muscle of MI + vehicle mice compared with sham + vehicle mice and was significantly decreased by Honokiol treatment (Figure 4B). The acetylation of lysine residues within mitochondrial proteins is regulated via acetylation by mitochondrial acetyltransferase and deacetylation by SIRT3 [25]. General control of amino acid synthesis 5 like 1 (GCN5L1) is known as a mitochondrial acetyltransferase and to be associated with the acetylation of various mitochondrial proteins, and it has been reported to act against SIRT3. SOD2 acetylation was increased in the hearts of high‐fat‐diet fed mice, but this increase in SOD2 acetylation did not occur in cardiac‐specific GCN5L1‐deleted mice, which do not have alterations in SIRT3 [26]. There was no significant difference in GCN5L1 between sham + vehicle and MI + vehicle (Figure S9). Therefore, it was suggested that GCN5L1 plays a minimal role in the increase in SOD2 acetylation in the skeletal muscle of MI mice.

We also evaluated SIRT3 expression and SOD2 acetylation levels in the heart. SIRT3 expression was decreased in the MI + vehicle mice compared with sham + vehicle, but SOD2 acetylation levels did not change significantly between the two groups (Figure S10). In addition, Honokiol treatment did not change SIRT3 expression and SOD2 acetylation (Figure S10). These results may be associated with the fact that Honokiol, which was started 2 weeks after surgery, did not change cardiac function.

Acetylation of SOD2 reduces SOD2 activity, which is thought to increase mitochondrial ROS production [15, 16]. Therefore, we measured H_2_O_2_ production in mitochondria isolated from the gastrocnemius muscle. It was increased in MI + vehicle mice compared with sham + vehicle mice, and it was inhibited in MI + Honokiol mice (Figure 4C). We also measured aconitase activity. Aconitase, an enzyme in the mitochondrial tricarboxylic acid (TCA) cycle that contains an iron–sulfur cluster in its active center, is known to be directly affected by ROS produced in mitochondria, resulting in a decrease in its activity [27]. It was decreased in MI + vehicle mice compared with sham + vehicle mice, and it was attenuated in MI + Honokiol mice (Figure 4D). On the other hand, there were no significant differences in expressions of acrolein and 4‐hydroxynonenal (4‐HNE) and the concentration of malondialdehyde among the three groups (Figure S11). These results suggest that the decreased SOD2 activity in skeletal muscle mitochondria increases mitochondrial ROS production and directly impairs mitochondrial function but does not lead to lipid peroxidation.

### 3.6 AAV9‐Mediated Overexpression of SIRT3 in Skeletal Muscle Improved the Exercise Capacity of MI Mice

**FIGURE 5:**
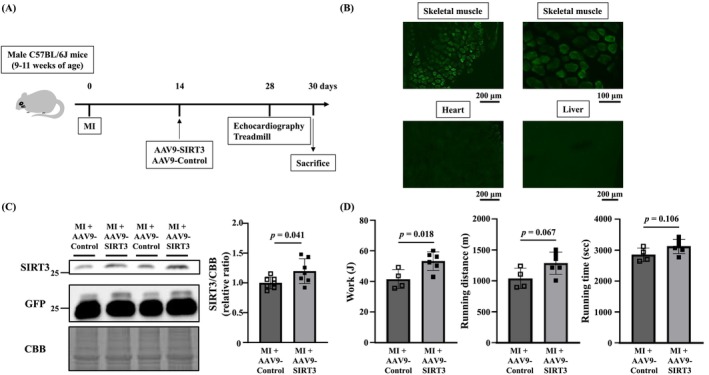
Overexpression of SIRT3 in skeletal muscle ameliorates reduced exercise capacity in MI mice. (A) Experimental protocol for AAV9 injection to MI mice. (B) Representative images of GFP expression in MI + AAV9‐Control mice in gastrocnemius muscle (low‐magnification, upper‐left), gastrocnemius muscle (high‐magnification, upper‐right), heart (bottom‐left), and liver (bottom‐right). (C) Representative western blots (left) and summary data (right) of SIRT3 in the gastrocnemius muscle of MI + AAV9‐Control (*n* = 7) and MI + AAV9‐SIRT3 mice (*n* = 7). (D) Summary data of work, running distance and running time of MI + AAV9‐Control (*n* = 7) and MI + AAV9‐SIRT3 mice (*n* = 7). Data are shown as the mean ± SD *p* values were calculated by the unpaired Student *t*‐test. AAV9, adeno‐associated virus serotype 9; SIRT3, sirtuin 3; GFP, green‐fluorescence protein; CBB, Coomassie Brilliant Blue; MI, myocardial infarction.

To further investigate whether the muscle‐specific increase in SIRT3 could improve the exercise capacity of MI mice, mice were treated with either AAV9‐SIRT3 or AAV9‐Control. Fluorescence microscopic observations confirmed GFP protein expression in the gastrocnemius muscle but not in the heart or liver (Figure 5B). SIRT3 expression was significantly increased in MI + AAV9‐SIRT3 mice compared with MI + AAV9‐Control mice (Figure 5C). Furthermore, the overexpression of SIRT3 improved exercise capacity (Figure 5D) without altering cardiac function and lung congestion (Figure S12).

## 4 Discussion

**FIGURE 6:**
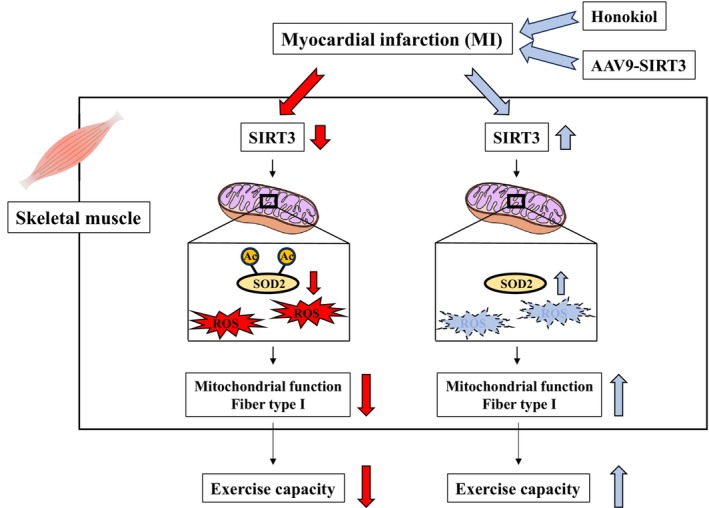
Schematic diagram of the potential roles of SIRT 3 and acetylated SOD2 in the skeletal muscle of MI mice and a possible therapeutic target for SIRT3 activator.

A summary of the findings of this study is shown in Figure 6. This study clearly showed that treatment with Honokiol, an activator of SIRT3, improved the exercise capacity of MI mice. This was owing to the reduction in ROS production and improvement of mitochondrial function in skeletal muscles via attenuation of SOD2 acetylation by Honokiol. In addition, the exercise capacity of MI mice was improved by skeletal muscle‐specific SIRT3 overexpression using AAV9. We believe that the present results will lead to the development of a therapy for improving exercise capacity in HF patients, which targets SIRT3 activation and acetylation reduction.

The decreased exercise capacity in HF is not only an indicator of the severity of the disease, but also an important factor in determining prognosis and quality of life [1]. Therefore, the development of drug treatments that improve exercise capacity is of great significance. In this study, Honokiol treatment improved the exercise capacity, especially the aerobic capacity, of a mouse model of HF after MI (Figure 2). The effect of Honokiol on exercise capacity was thought to be owing to an improvement in skeletal muscle mitochondrial function. In general, it is believed that there is no association between decreased exercise capacity in patients with HF and impaired cardiac function at rest or disturbed central circulation [1, 28, 29]. However, a positive effect of Honokiol on cardiac function cannot be completely excluded in our experiments. The administration of Honokiol to MI mice did not affect the parameters of echocardiography or organ weights (Figure S4), but we were unable to evaluate the cardiac reserve of mice during exercise owing to technical reasons. Furthermore, it has been reported that administration of Honokiol for 4 weeks attenuated cardiac hypertrophy, improved cardiac function, and reduced interstitial fibrosis in mice after transverse aortic constriction [19]. Although the reasons for the differences between this previous study and our results are unclear, they may be owing to the different HF models used (MI in our study vs. transverse aortic constriction in the previous study) and the duration of Honokiol treatment (2 weeks in our study vs. 4 weeks in the previous study).

Honokiol has been shown to have antitumour, anti‐inflammatory, nerve protection and antioxidant effects [24, 30, 31]. These effects are each thought to occur via SIRT3‐dependent or ‐independent molecular mechanisms. In our present research using C2C12 cultured skeletal muscle cells, the effect of Honokiol was found to be via SIRT3 (Figure 1). Furthermore, our results showing the improvement of exercise capacity by AAV9‐mediated SIRT3 overexpression also supported the importance of SIRT3 (Figure 5). On the other hand, Honokiol can also activate SIRT1 [32]. In the present study, we analysed the expression level of SIRT1, but it was not altered by Honokiol administration (Figure S7). Therefore, we assume that SIRT1 was not involved in the effects of Honokiol in the present study.

SOD2 is located in mitochondria, the main source of ROS in organisms, and plays an important role in eliminating ROS. However, an increase in SOD2 protein expression on its own has a weak ability to reduce intracellular ROS [33]. Acetylation of the lysine residues of SOD2, which is a highly preserved enzyme activity center, suppresses SOD2 activity [15]. On the other hand, SOD2 is activated by SIRT3‐dependent deacetylation, which reduces intracellular ROS [16, 34]. The SIRT3 expression in skeletal muscle was decreased and SOD2 acetylation was increased in MI mice (Figure 4A,B). This led to an increase in ROS production in skeletal muscle (Figure 4C,D). In a mouse model of angiotensin II‐induced hypertension, it was shown that aortic SOD2 acetylation had an inverse correlation with aortic SOD2 activity and a direct correlation with hypertension severity [35]. Furthermore, in mice undergoing caloric restriction, oxidative stress was reduced by SIRT3‐mediated SOD2 deacetylation and activation [33]. These results support our present results.

The acetylation of mitochondrial proteins extracted from the skeletal muscle of MI mice was increased, but Honokiol treatment did not decrease this acetylation despite its normalization of SIRT3 expression levels (Figure 4A and Figure S8). The reason for this is not clear, but it may be associated with the fact that Honokiol treatment was started 2 weeks after MI surgery and was administered for 4 weeks. This protocol may not reduce the acetylation of some mitochondrial proteins. Many mitochondrial proteins are known to undergo acetylation modifications, and their functions are regulated [36]. These contain proteins associated with mitochondrial metabolism, such as lipid metabolism, electron transfer/oxidative phosphorylation, the TCA cycle and amino acid metabolism [37]. In fact, our previous acetylome analysis demonstrated that various enzymes involved in fatty acid metabolism, the TCA cycle, and electron transfer were strongly acetylated in the skeletal muscle of mice after MI [17]. In the present study, we could not clarify whether the favourable effects of Honokiol on exercise capacity were mediated by the deacetylation of mitochondrial proteins other than SOD2.

Our present study clearly showed the effects of SIRT3 activation on the skeletal muscle abnormalities of MI mice. To date, several drugs that stimulate SIRT3 expression and enhance SIRT3 activation have been identified [23]. Natural compounds, molecules, and hormones that have attracted attention as pharmaceuticals have been studied as SIRT3 activators, but Honokiol is the representative of natural compounds that activate SIRT3 and is the most researched. In recent years, the development of more specific SIRT3 activators is being pursued by compound screening using the SIRT3‐SOD2K68AcK (SOD2 acetylated at Lys 68) complex reaction system and screening by molecular bonds based on the SOD2 structure [38]. We expect that treatments for reduced exercise capacity and skeletal muscle abnormalities in HF patients based on SIRT3 activation and reduced mitochondrial protein acetylation will be developed in the future.

There are several limitations to this study that should be mentioned. First, functional measurements of skeletal muscles isolated from mice were not evaluated in this study. The importance of skeletal muscle contribution to exercise capacity can be demonstrated by assessing the properties of skeletal muscle itself. This is a crucial issue that remains to be addressed in the future. Second, the reason for the discrepancy in the results of direct ROS measurement (Figure 4C) and lipid peroxidation assessment (Figure S11) is unclear, but it may be that the severity of skeletal muscle abnormalities in the model used in this study was mild. In general, enhanced ROS production in mitochondria reduces mitochondrial function and aconitase activity, which are in proximity to the source of ROS and susceptible to ROS damage. On the other hand, lipid peroxidation by highly damaging ROS, that is, hydroxyl radicals, occurs at the later stages of cell injury [39], and has not yet occurred in the skeletal muscle in this study. These are consistent with the absence of abnormalities in the number and morphology of mitochondria (Figure 3C,D and Figure S7). Third, the mice used in this study were around 10 weeks of age. Generally, in studies using models of HF after MI, mice of a similar age have been used to study the molecular mechanisms of HF. However, considering that the majority of patients who develop MI are elderly, it is necessary to conduct research using ageing models. However, the experimental design is complex because both factors of ageing and MI are related, and we could not determine the contribution of ageing in this study. This important point is considered to be a future challenge. Finally, the experiments using C2C12 cells were unable to reproduce an in vivo model. Ideally, an experimental design would be required to establish a cell model that mimics skeletal muscle abnormalities by applying some kind of stimulus. This is believed to be largely due to the fact that the causes of skeletal muscle abnormalities in HF have not been clarified. The main purpose of the in vitro study was to verify the effects of Honokiol in vivo, and the cell experiments demonstrated that the effects of Honokiol are indeed mediated by SIRT3.

The SIRT3 activator Honokiol improved exercise capacity in a myocardial infarction model mice with heart failure by improving mitochondrial function in their skeletal muscle through the reduction of SOD2 acetylation. Our results demonstrate that SIRT3 activation may be a novel therapeutic target for improving exercise capacity in patients with heart failure.

## Ethics Statement

The authors certify that they comply with the ethical guidelines for authorship and publishing of the *Journal of Cachexia, Sarcopenia and Muscle* [40].

## Conflicts of Interest

The authors declare no conflicts of interest.

## Supporting information


**Figure S1.** SIRT4 and SIRT5 expressions in the skeletal muscle Representative western blots and summary data of SIRT4 and SIRT 5 in the gastrocnemius muscle of sham + vehicle (*n* = 6) and MI + vehicle (*n* = 6). The blots were normalized to the nonspecific bands of CBB‐stained gel. Data are shown as the mean ± SD *p* values were calculated by the unpaired Student *t*‐test. MI, myocardial infarction; CBB, SIRT4, sirtuin 4; SIRT5, sirtuin 5; Coomassie Brilliant Blue.

**Figure S2.** Acetylated lysine in the mitochondrial lysates (A) Representative western blot (left) and summary data (right) of acetylated lysine in the mitochondrial lysates from C2C12 myotubes treated with vehicle (*n* = 7) and Honokiol (*n* = 7). (B) Representative western blot (left) and summary data (right) of acetylated lysine in the mitochondrial lysates from C2C12 myotubes treated with vehicle (*n* = 4) and Honokiol (*n* = 4) with transfection of SIRT3 siRNA. Results were normalized to nonspecific bands of the CBB‐stained gel. Data are shown as the mean ± SD *p* values were calculated by the unpaired Student *t*‐test. CBB, Coomassie Brilliant Blue.

**Figure S3.** Echocardiographic data of sham and MI mice before treatment with vehicle or Honokiol 2 weeks after surgery. Summary data of left ventricular end‐diastolic diameter (A), left ventricular end‐systolic diameter (B), fractional shortening (C), and heart rate (D) in sham + vehicle (*n* = 8), sham + Honokiol (*n* = 7), MI + vehicle (*n* = 8) and MI + Honokiol mice (*n* = 6). Data are shown as the mean ± SD *p* values of the main effect for each factor and interaction effect between two factors were calculated by two‐way ANOVA with the factors of MI and Honokiol. MI, myocardial infarction.

**Figure S4.** Echocardiographic data of sham and MI mice treated with vehicle or Honokiol 4 weeks after surgery Representative M‐mode echocardiographic images (A) and summary data of left ventricular end‐diastolic diameter (B), left ventricular end‐systolic diameter (C), fractional shortening (D), and heart rate (E) in sham + vehicle (*n* = 8), sham + Honokiol (*n* = 7), MI + vehicle (*n* = 8), and MI + Honokiol mice (*n* = 6). Data are shown as the mean ± SD *p* values of the main effect for each factor and interaction effect between two factors were calculated by two‐way ANOVA with the factors of MI and Honokiol. ANOVA with the factors of MI and Honokiol. MI, myocardial infarction.

**Figure S5.** Organ weights in MI mice and sham mice treated with vehicle or Honokiol Summary data of heart weight/body weight (A), and lung weight/body weight (B) in sham + vehicle (*n* = 8), sham + Honokiol (*n* = 7), MI + vehicle (*n* = 8), and MI + Honokiol mice (*n* = 6). Data are shown as the mean ± SD *p* values of the main effect for each factor, and interaction effect between two factors were calculated by two‐way ANOVA with the factors of MI and Honokiol. MI, myocardial infarction.

**Figure S6** Immunofluorescence staining and gene expression of MHC. (A) Summary data of the proportion of Type IIa (top) and Type IIb (bottom) fibres in the gastrocnemius muscle of sham + vehicle (*n* = 5), MI + vehicle (*n* = 5) and MI + Honokiol (*n* = 5) to the total fibres. (B) Summary data of gene expression of *Myh7*, *Myh2*, *Myh1* and *Myh4* in the gastrocnemius muscle of sham + vehicle (*n* = 8), MI + vehicle (*n* = 7) and MI + Honokiol mice (*n* = 8). The expression of each gene was normalized to that of *18S*. Data are shown as the mean ± SD *p* values were calculated by one‐way ANOVA followed by the Tukey post hoc test. MI, myocardial infarction.

**Figure S7.** Expression of proteins associated with mitochondrial biogenesis, fission, fusion, and mitophagy Representative western blots (A) and summary data (B) of PGC‐1α, SIRT1, Mitofusin1 Mitofusin2, Optic atrophy1, DRP1, PINK1, and Parkin in the gastrocnemius muscle of sham + vehicle (*n* = 6), MI + vehicle (*n* = 6) and MI + Honokiol (*n* = 6). The blots were normalized to the nonspecific bands of CBB‐stained gel. Data are shown as the mean ± SD *p* values were calculated by one‐way ANOVA followed by the Tukey post hoc test. MI, myocardial infarction; PGC‐1α, peroxisome proliferator‐activated receptor γ coactivator‐1 α; SIRT1, sirtuin 3; DRP1, dynamin related protein 1; PINK1, PTEN‐ induced serine/threonine kinase1; CBB, Coomassie Brilliant Blue.

**Figure S8.** Acetylated lysine in the mitochondrial lysates Representative western blot (left) and summary data (right) of acetylated lysine in the mitochondrial lysates from the skeletal muscle of sham + vehicle (*n* = 7), MI + vehicle (*n* = 8), and MI + Honokiol mice (*n* = 8). Results were normalized to non‐specific bands of the CBB‐stained gel. Data are shown as the mean ± SD *p* values were calculated by one‐way ANOVA followed by the Tukey *post hoc* test. MI, myocardial infarction; CBB, Coomassie Brilliant Blue.

**Figure S9.** GCN5L1 expression in sham mice and MI mice Representative western blots and summary data of GCN5L1 in the gastrocnemius muscle of sham + vehicle (*n* = 5) and MI + vehicle (*n* = 4). Results were normalized to the nonspecific bands of CBB‐stained gel. Data are shown as the mean ± SD *p* values were calculated by the unpaired Student *t*‐test. MI, myocardial infarction; GCN5L1, general control of amino acid synthesis 5 like 1; CBB, Coomassie Brilliant Blue.

**Figure S10.** SIRT3 expression and acetylated SOD2 in the heart Representative western blots (left) and summary data (right) of SIRT3 and acetylated SOD2 in the Heart of sham + vehicle (*n* = 4), MI + vehicle (*n* = 4), and MI + Honokiol mice (*n* = 4). SIRT3 was normalized to non‐specific bands of the CBB‐stained gel. Acetylated SOD2 was normalized to total SOD2. Data are shown as the mean ± SD *p* values were calculated by one‐way ANOVA followed by the Tukey post hoc test. MI, myocardial infarction; SIRT3, sirtuin 3, SOD2, superoxide dismutase 2; CBB, Honokiol mice (*n* = 8). Acrolein and 4‐HNE were normalized to nonspecific bands of the CBB‐stained gel. Data are shown as the mean ± SD. *p* values were calculated by one‐way ANOVA followed by the Tukey post hoc test. MI, myocardial infarction; AAV9, adeno‐associated virus serotype 9; SIRT3, sirtuin 3; MI, myocardial infarction.

**Figure S11.** Lipid peroxidation in the skeletal muscle after MI. (A) Representative western blots (top) and summary data (bottom) of acrolein in the gastrocnemius muscle of sham + vehicle (*n* = 7), MI + vehicle (*n* = 7), and MI + Honokiol mice (*n* = 7). (B) Representative western blots (top) and summary data (bottom) of 4‐HNE in the gastrocnemius muscle of sham + vehicle (*n* = 3), MI + vehicle (*n* = 3) and MI + Honokiol mice (*n* = 3). (C) Summary data of malondialdehyde in the gastrocnemius muscle of sham + vehicle (*n* = 8), MI + vehicle (*n* = 8), and MI + Honokiol mice (*n* = 8). Acrolein and 4‐HNE were normalized to non‐specific bands of the CBB‐stained gel. Data are shown as the mean ± SD *p* values were calculated by one‐way ANOVA followed by the Tukey post hoc test. MI, myocardial infarction; CBB, Coomassie Brilliant Blue; 4‐HNE, 4‐hydroxynonenal.

**Figure S12.** Echocardiographic data and organ weight in MI mice treated with AAV9‐Control or AAV‐SIRT3. Summary data of body weight (A), and gastrocnemius weight/body weight, heart weight/body weight and lung weight/body weight (B), and left ventricular end‐diastolic diameter, left ventricular end‐systolic diameter and fractional shortening (C), and heart rate (D) in MI + AAV9‐Control (*n* = 4) and MI + AAV9‐SIRT3 mice (*n* = 6). Data are shown as the mean ± SD *p* values were calculated by the unpaired Student *t*‐test. AAV9, adeno‐associated virus serotype 9; SIRT3, sirtuin 3; MI, myocardial infarction.

**Figure S13.** Treadmill system and exercise protocol The treadmill system (A) and exercise protocol (B) were shown. Running distance (meters) was measured as the distance during exercise in a direction along the treadmill. Run time (seconds) was expressed as the time from the end of warm‐up to exhaustion. Vertical distance (meters) was calculated by multiplying the running distance (meters) by sin 10°. The work (Joules) performed by the mice during exercise was calculated by vertical distance (meters) by body weight (kilograms) and then by standard gravitational acceleration (meters per second squared). *g*, standard gravitational acceleration.

**Data S1.** Supplementary Information.
