# Supplementary figures and images for: Reduction in Acetylation of Superoxide Dismutase 2 in Skeletal Muscle Improves Exercise Capacity in Mice With Heart Failure
Source: J Cachexia Sarcopenia Muscle. 2025 Jun 13;16(3):e13850. doi: 10.1002/jcsm.13850 (PMC12163645; doi:10.1002/jcsm.13850)

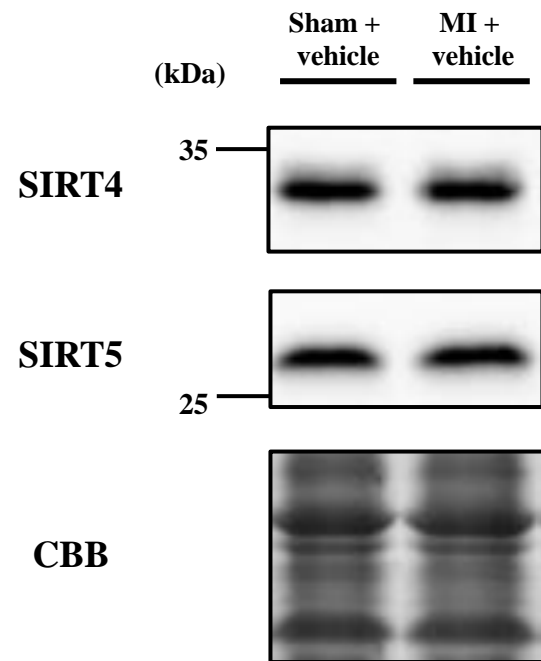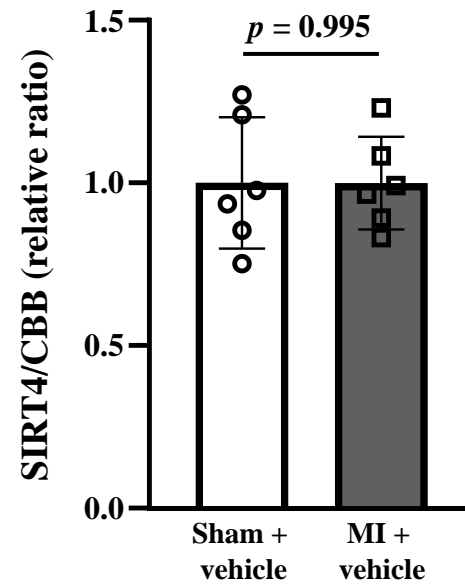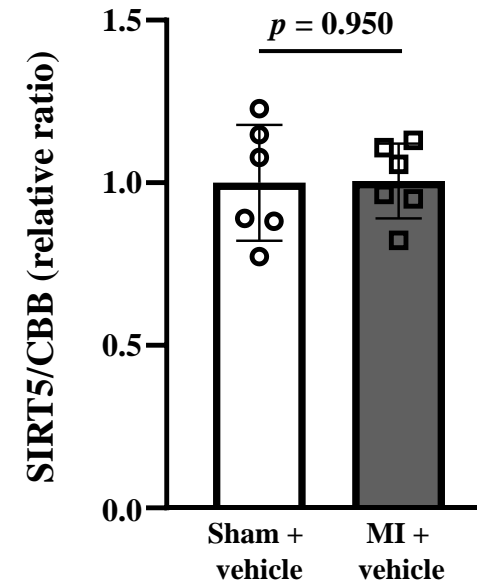

Figure S1

Supplement: Supplementary file 1 — Figure S1. SIRT4 and SIRT5 expressions in the skeletal muscle Representative western blots and summary data of SIRT4 and SIRT 5 in the gastrocnemius muscle of sham + vehicle (n = 6) and MI + vehicle (n = 6). The blots were normalized to the nonspecific bands of CBB‐stained gel. Data are shown as the mean ± SD p values were calculated by the unpaired Student t‐test. MI, myocardial infarction; CBB, SIRT4, sirtuin 4; SIRT5, sirtuin 5; Coomassie Brilliant Blue. [file JCSM-16-e13850-s003.pdf]

(A)

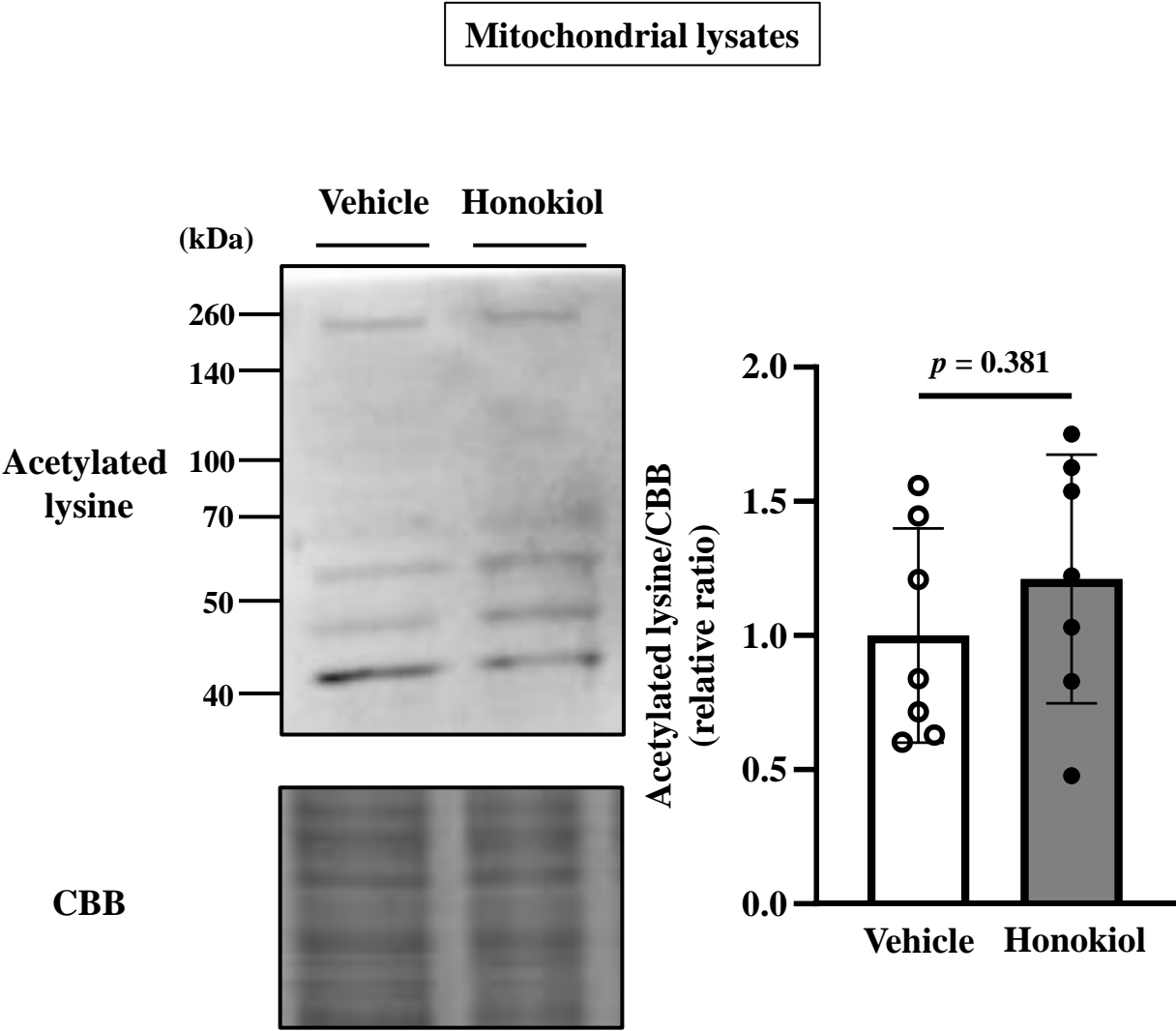

(B)

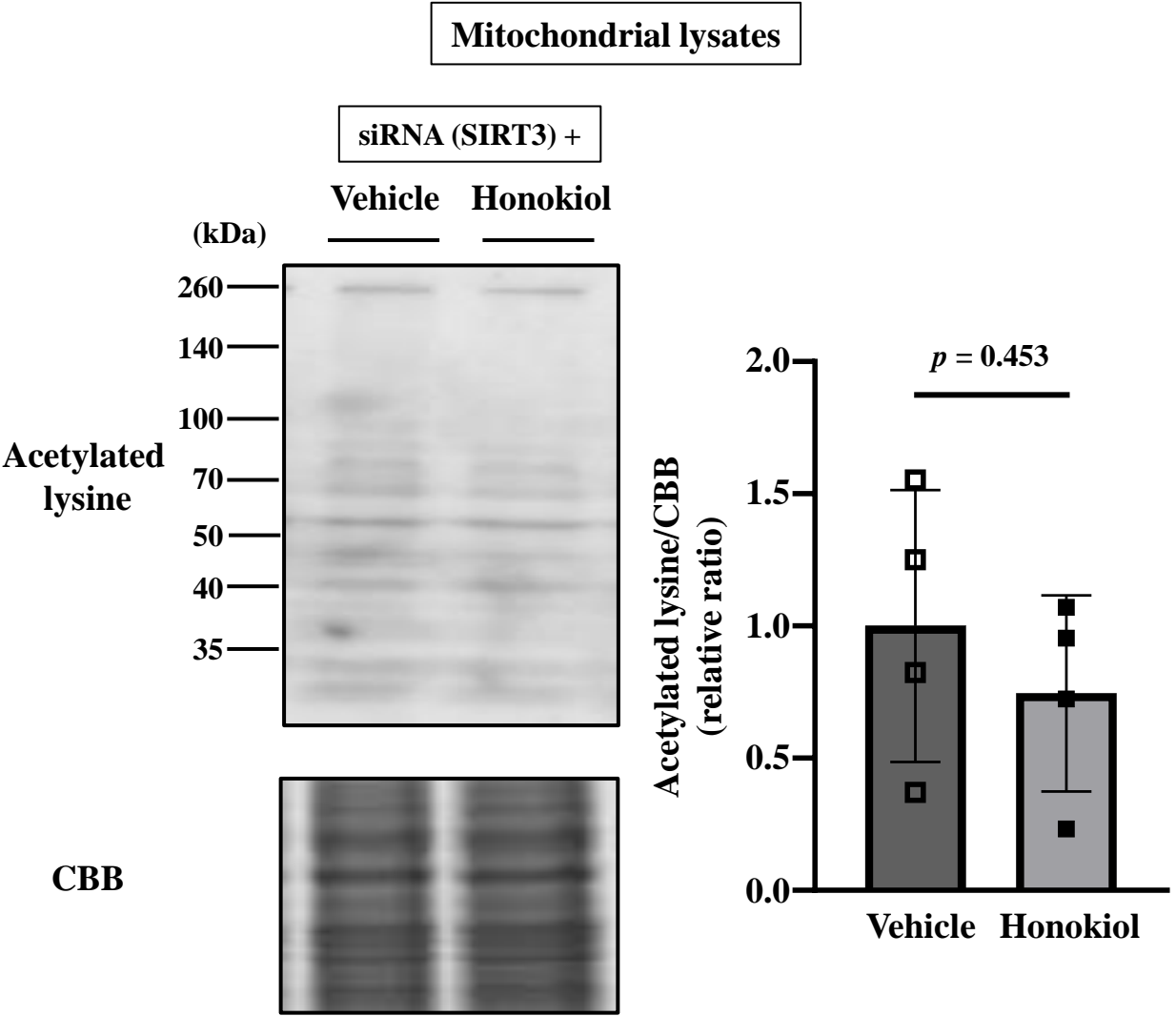

Figure S2

Supplement: Supplementary file 2 — Figure S2. Acetylated lysine in the mitochondrial lysates (A) Representative western blot (left) and summary data (right) of acetylated lysine in the mitochondrial lysates from C2C12 myotubes treated with vehicle (n = 7) and Honokiol (n = 7). (B) Representative western blot (left) and summary data (right) of acetylated lysine in the mitochondrial lysates from C2C12 myotubes treated with vehicle (n = 4) and Honokiol (n = 4) with transfection of SIRT3 siRNA. Results were normalized to nonspecific bands of the CBB‐stained gel. Data are shown as the mean ± SD p values were calculated by the unpaired Student t‐test. CBB, Coomassie Brilliant Blue. [file JCSM-16-e13850-s006.pdf]

2 weeks after surgery

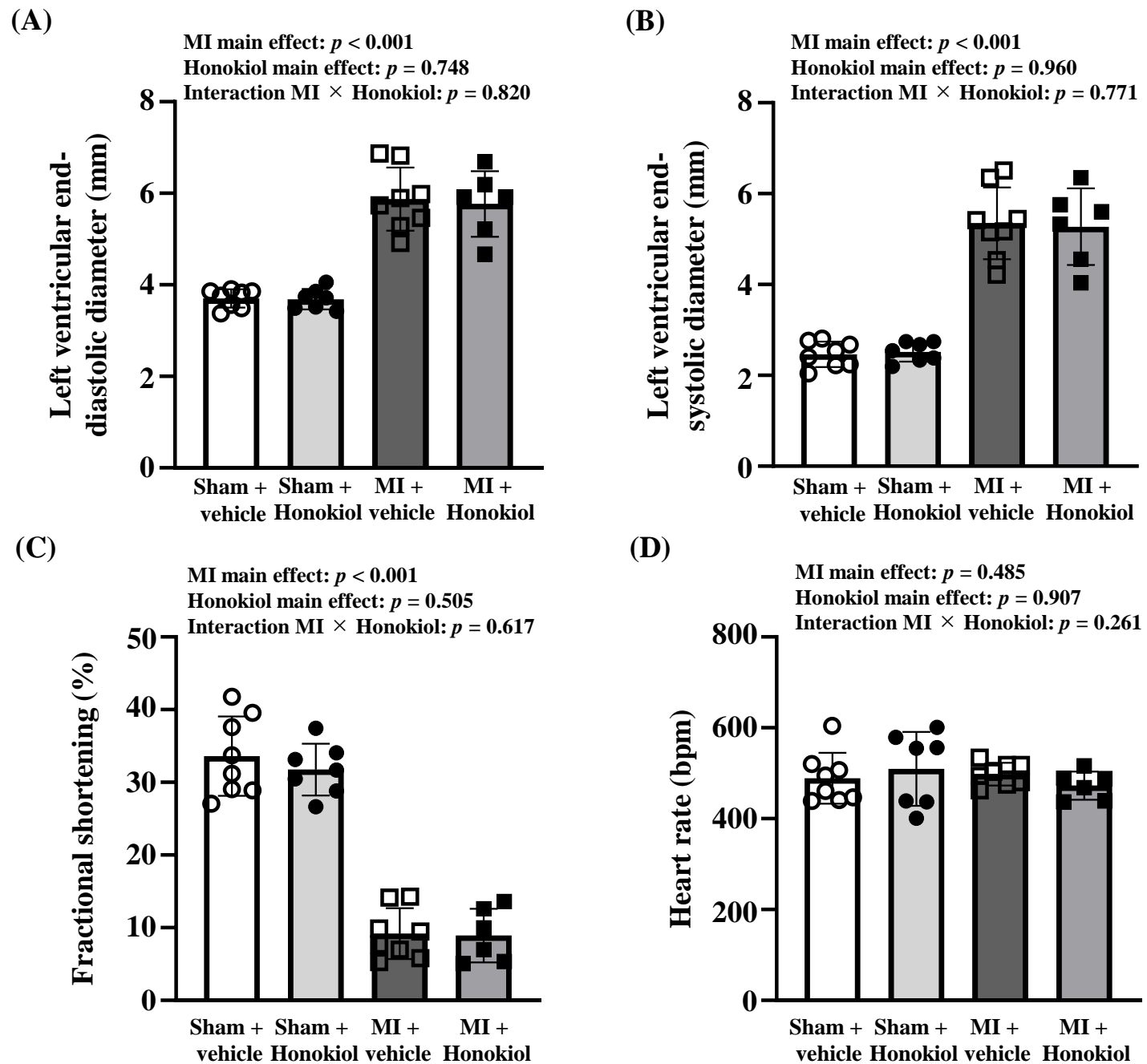

Figure S3

Supplement: Supplementary file 3 — Figure S3. Echocardiographic data of sham and MI mice before treatment with vehicle or Honokiol 2 weeks after surgery. Summary data of left ventricular end‐diastolic diameter (A), left ventricular end‐systolic diameter (B), fractional shortening (C), and heart rate (D) in sham + vehicle (n = 8), sham + Honokiol (n = 7), MI + vehicle (n = 8) and MI + Honokiol mice (n = 6). Data are shown as the mean ± SD p values of the main effect for each factor and interaction effect between two factors were calculated by two‐way ANOVA with the factors of MI and Honokiol. MI, myocardial infarction. [file JCSM-16-e13850-s014.pdf]

(A)

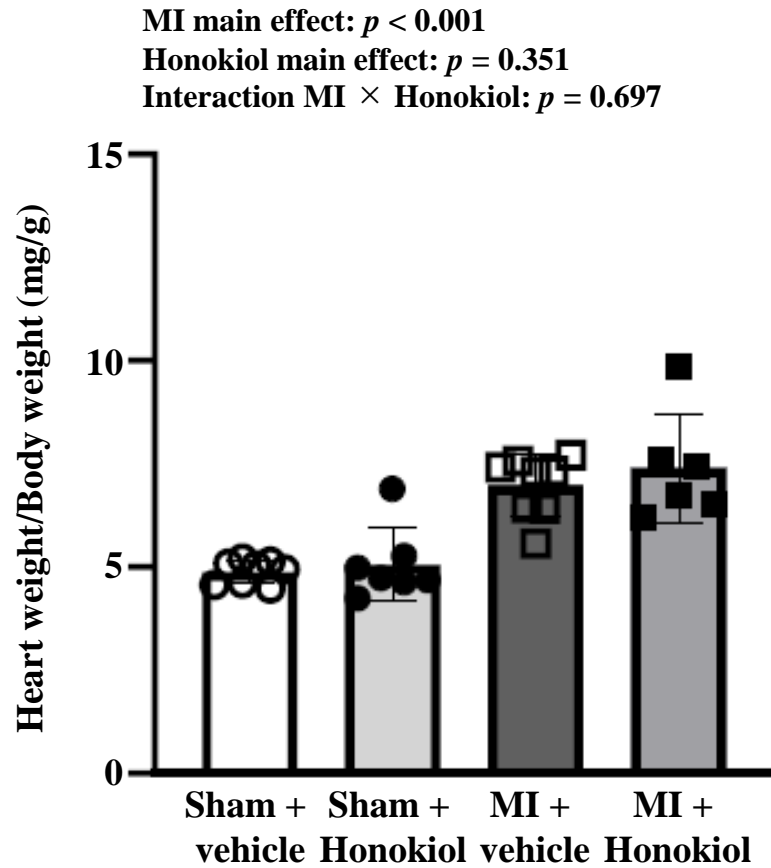

(B)

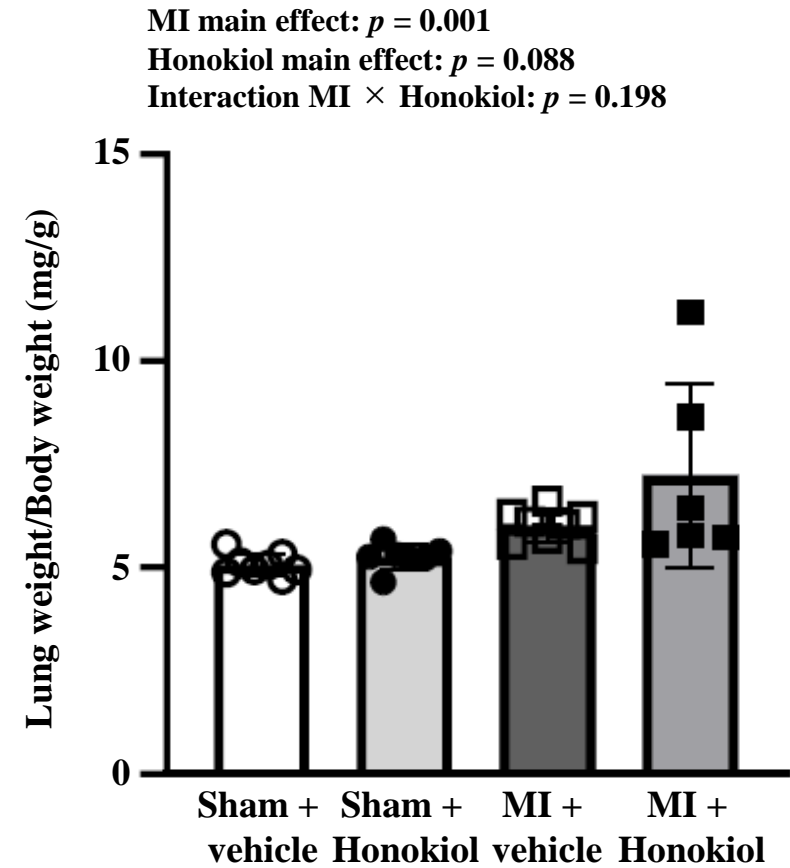

Supplement: Supplementary file 5 — Figure S5. Organ weights in MI mice and sham mice treated with vehicle or Honokiol Summary data of heart weight/body weight (A), and lung weight/body weight (B) in sham + vehicle (n = 8), sham + Honokiol (n = 7), MI + vehicle (n = 8), and MI + Honokiol mice (n = 6). Data are shown as the mean ± SD p values of the main effect for each factor, and interaction effect between two factors were calculated by two‐way ANOVA with the factors of MI and Honokiol. MI, myocardial infarction. [file JCSM-16-e13850-s005.pdf]

(A)

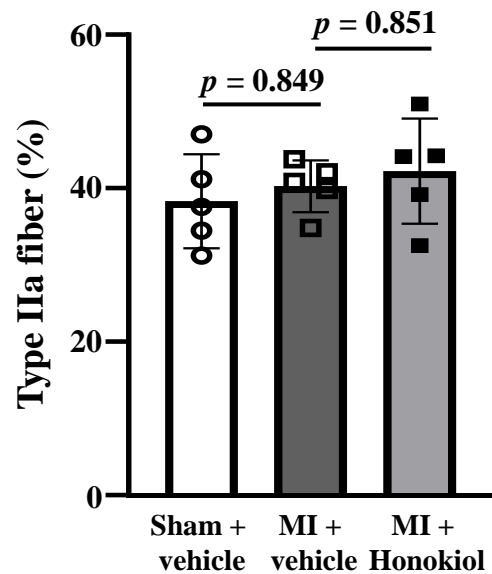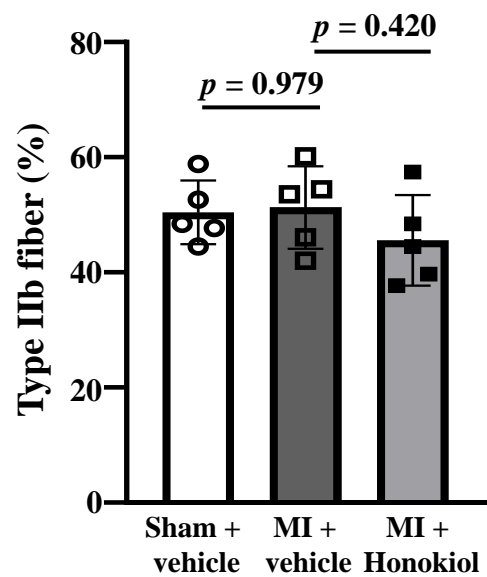

(B)

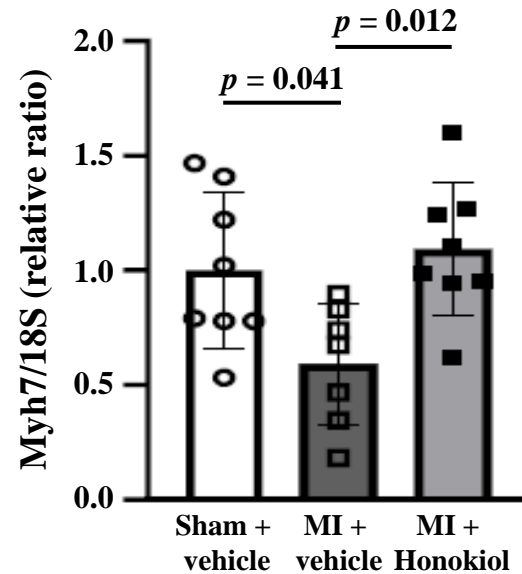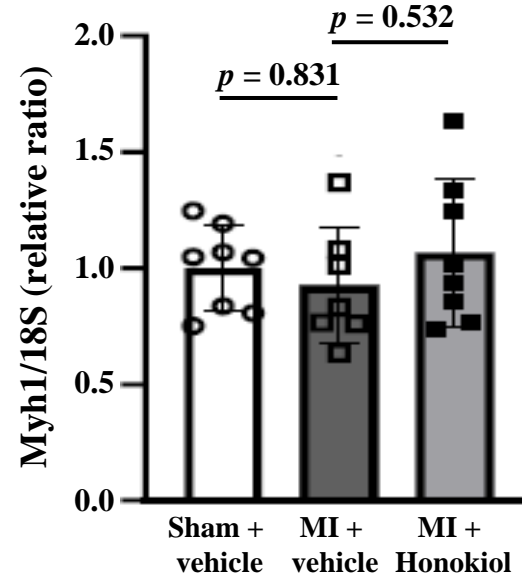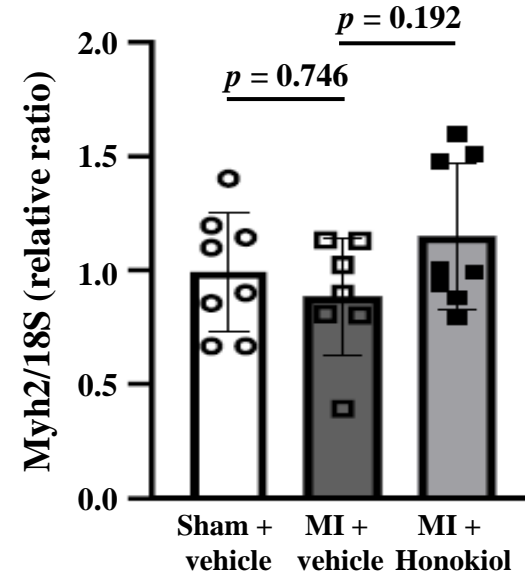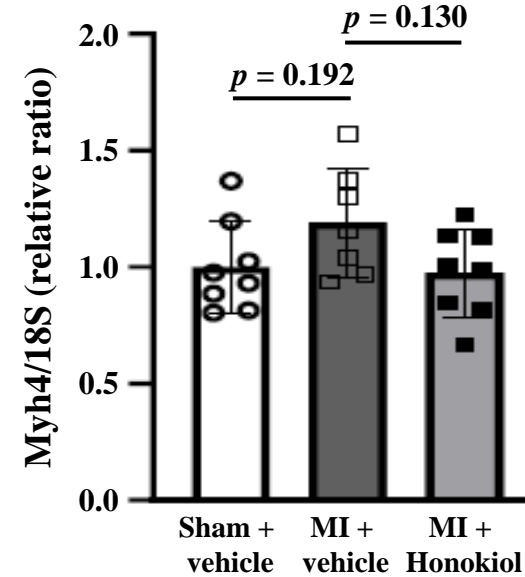

Figure S6

Supplement: Supplementary file 6 — Figure S6 Immunofluorescence staining and gene expression of MHC. (A) Summary data of the proportion of Type IIa (top) and Type IIb (bottom) fibres in the gastrocnemius muscle of sham + vehicle (n = 5), MI + vehicle (n = 5) and MI + Honokiol (n = 5) to the total fibres. (B) Summary data of gene expression of Myh7, Myh2, Myh1 and Myh4 in the gastrocnemius muscle of sham + vehicle (n = 8), MI + vehicle (n = 7) and MI + Honokiol mice (n = 8). The expression of each gene was normalized to that of 18S. Data are shown as the mean ± SD p values were calculated by one‐way ANOVA followed by the Tukey post hoc test. MI, myocardial infarction. [file JCSM-16-e13850-s007.pdf]

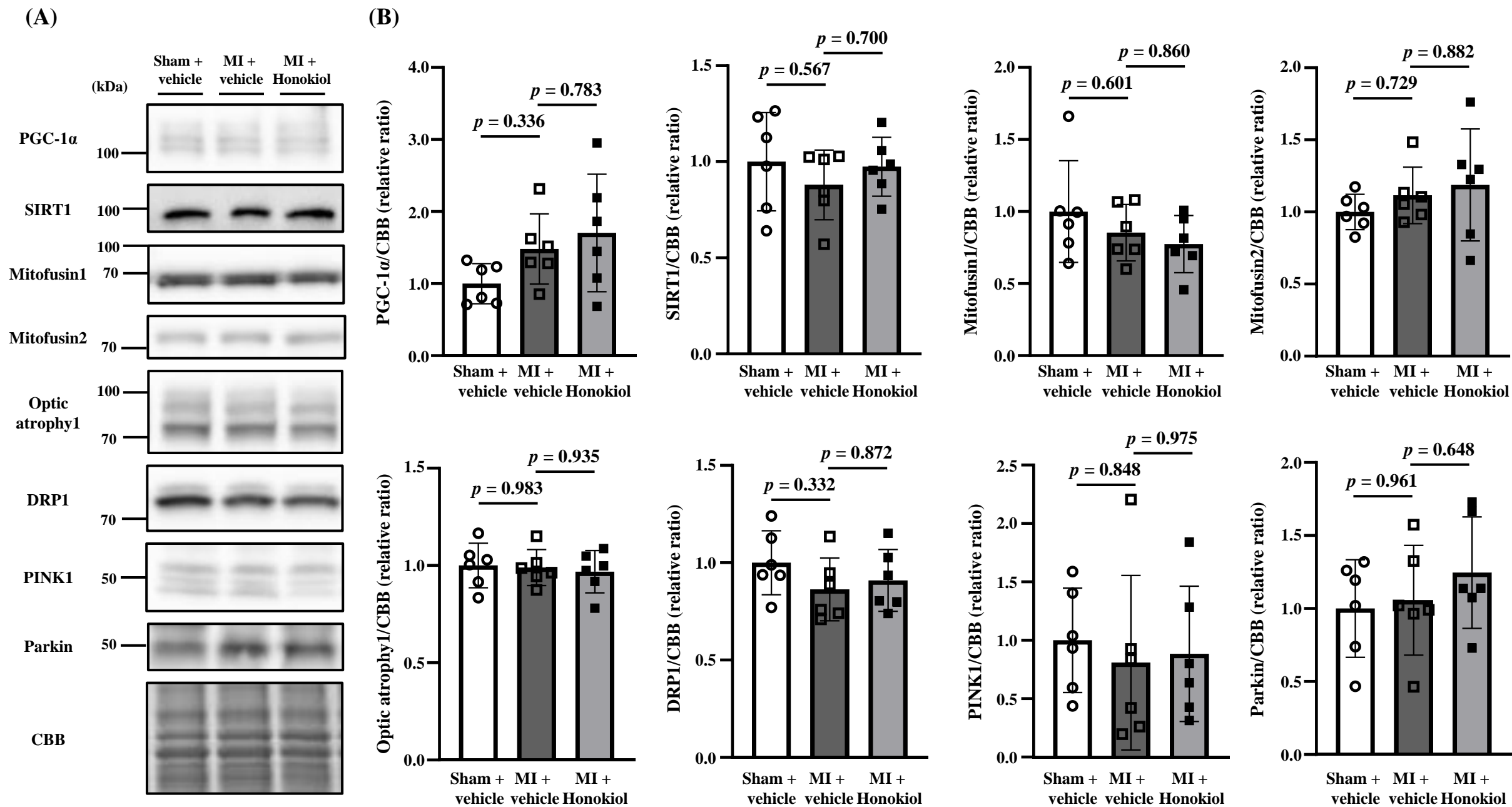

Figure S7

Supplement: Supplementary file 7 — Figure S7. Expression of proteins associated with mitochondrial biogenesis, fission, fusion, and mitophagy Representative western blots (A) and summary data (B) of PGC‐1α, SIRT1, Mitofusin1 Mitofusin2, Optic atrophy1, DRP1, PINK1, and Parkin in the gastrocnemius muscle of sham + vehicle (n = 6), MI + vehicle (n = 6) and MI + Honokiol (n = 6). The blots were normalized to the nonspecific bands of CBB‐stained gel. Data are shown as the mean ± SD p values were calculated by one‐way ANOVA followed by the Tukey post hoc test. MI, myocardial infarction; PGC‐1α, peroxisome proliferator‐activated receptor γ coactivator‐1 α; SIRT1, sirtuin 3; DRP1, dynamin related protein 1; PINK1, PTEN‐ induced serine/threonine kinase1; CBB, Coomassie Brilliant Blue. [file JCSM-16-e13850-s008.pdf]

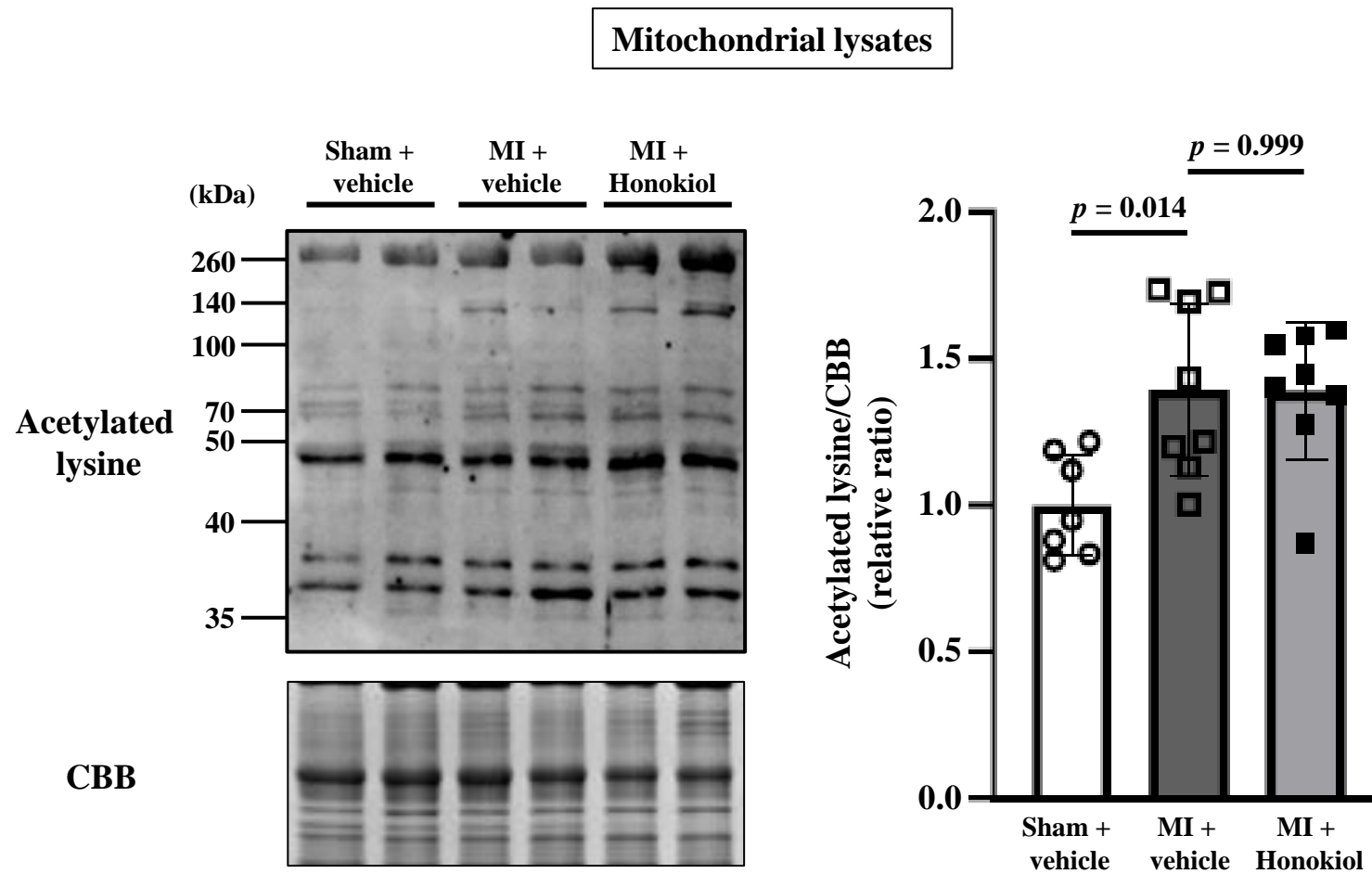

**Figure S8**

Supplement: Supplementary file 8 — Figure S8. Acetylated lysine in the mitochondrial lysates Representative western blot (left) and summary data (right) of acetylated lysine in the mitochondrial lysates from the skeletal muscle of sham + vehicle (n = 7), MI + vehicle (n = 8), and MI + Honokiol mice (n = 8). Results were normalized to non‐specific bands of the CBB‐stained gel. Data are shown as the mean ± SD p values were calculated by one‐way ANOVA followed by the Tukey post hoc test. MI, myocardial infarction; CBB, Coomassie Brilliant Blue. [file JCSM-16-e13850-s012.pdf]

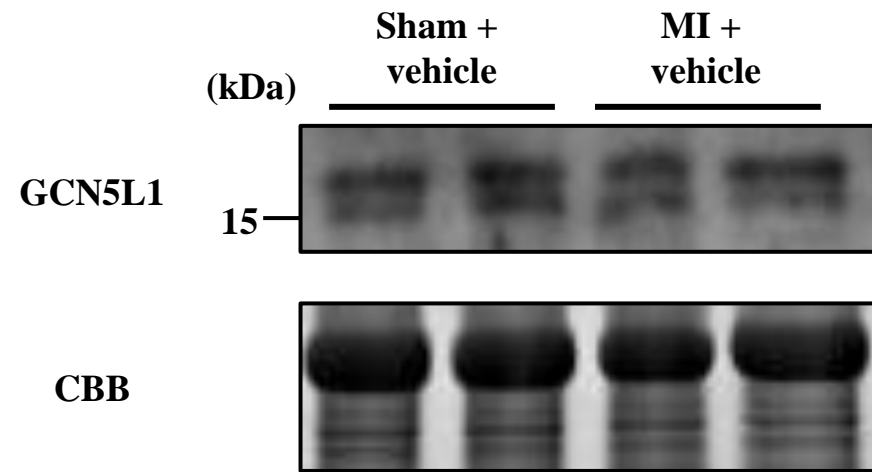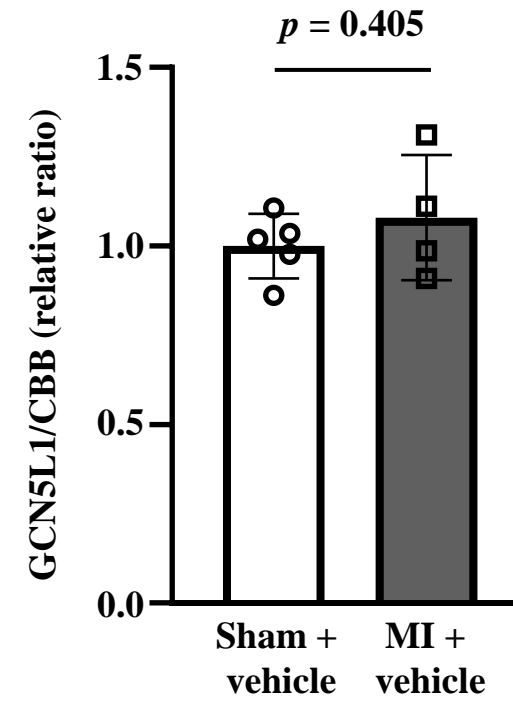

Figure S9

Supplement: Supplementary file 9 — Figure S9. GCN5L1 expression in sham mice and MI mice Representative western blots and summary data of GCN5L1 in the gastrocnemius muscle of sham + vehicle (n = 5) and MI + vehicle (n = 4). Results were normalized to the nonspecific bands of CBB‐stained gel. Data are shown as the mean ± SD p values were calculated by the unpaired Student t‐test. MI, myocardial infarction; GCN5L1, general control of amino acid synthesis 5 like 1; CBB, Coomassie Brilliant Blue. [file JCSM-16-e13850-s011.pdf]

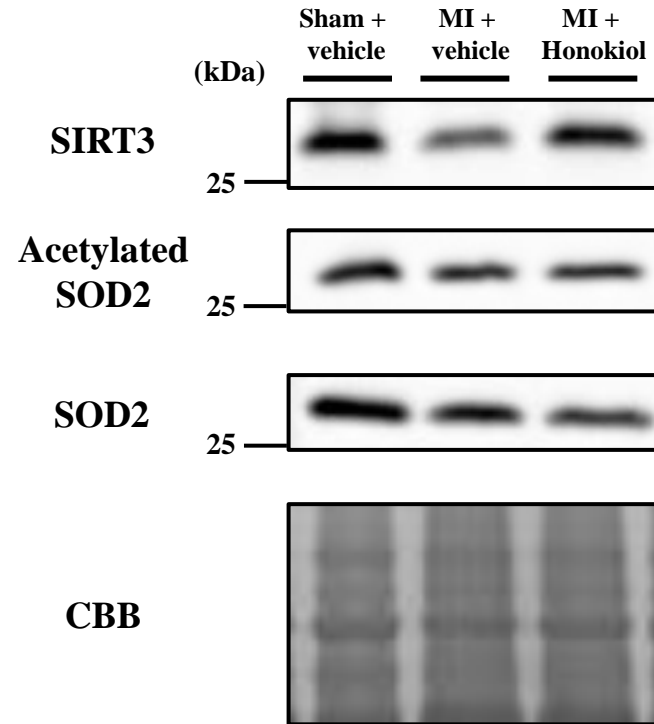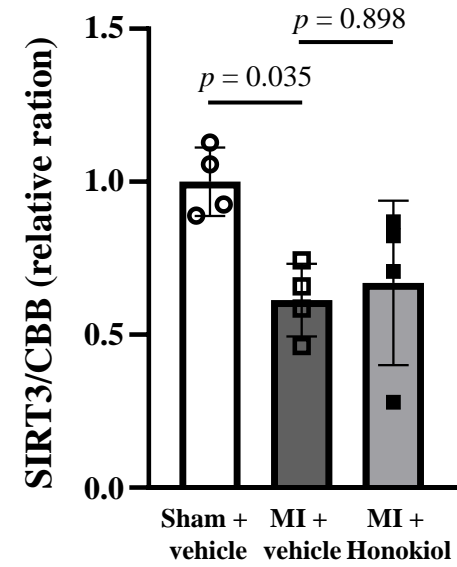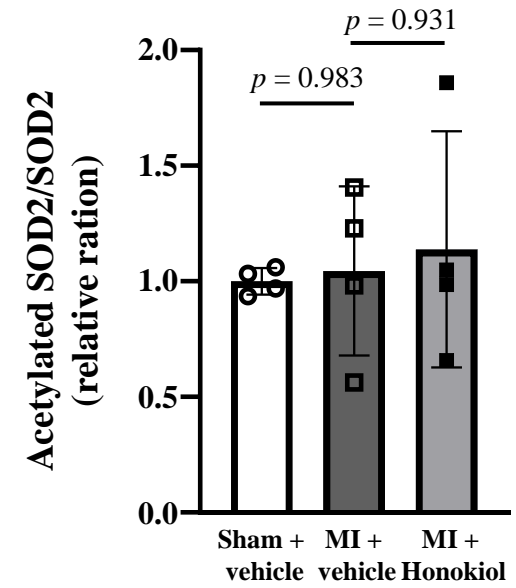

**Figure S10**

Supplement: Supplementary file 10 — Figure S10. SIRT3 expression and acetylated SOD2 in the heart Representative western blots (left) and summary data (right) of SIRT3 and acetylated SOD2 in the Heart of sham + vehicle (n = 4), MI + vehicle (n = 4), and MI + Honokiol mice (n = 4). SIRT3 was normalized to non‐specific bands of the CBB‐stained gel. Acetylated SOD2 was normalized to total SOD2. Data are shown as the mean ± SD p values were calculated by one‐way ANOVA followed by the Tukey post hoc test. MI, myocardial infarction; SIRT3, sirtuin 3, SOD2, superoxide dismutase 2; CBB, Honokiol mice (n = 8). Acrolein and 4‐HNE were normalized to nonspecific bands of the CBB‐stained gel. Data are shown as the mean ± SD. p values were calculated by one‐way ANOVA followed by the Tukey post hoc test. MI, myocardial infarction; AAV9, adeno‐associated virus serotype 9; SIRT3, sirtuin 3; MI, myocardial infarction. [file JCSM-16-e13850-s004.pdf]

(A)

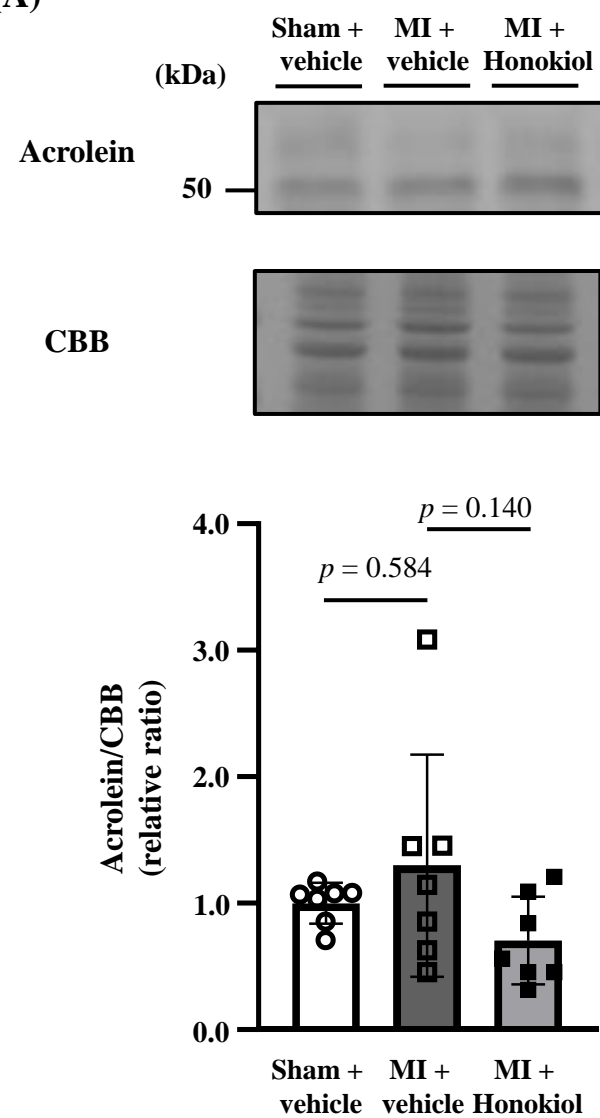

(B)

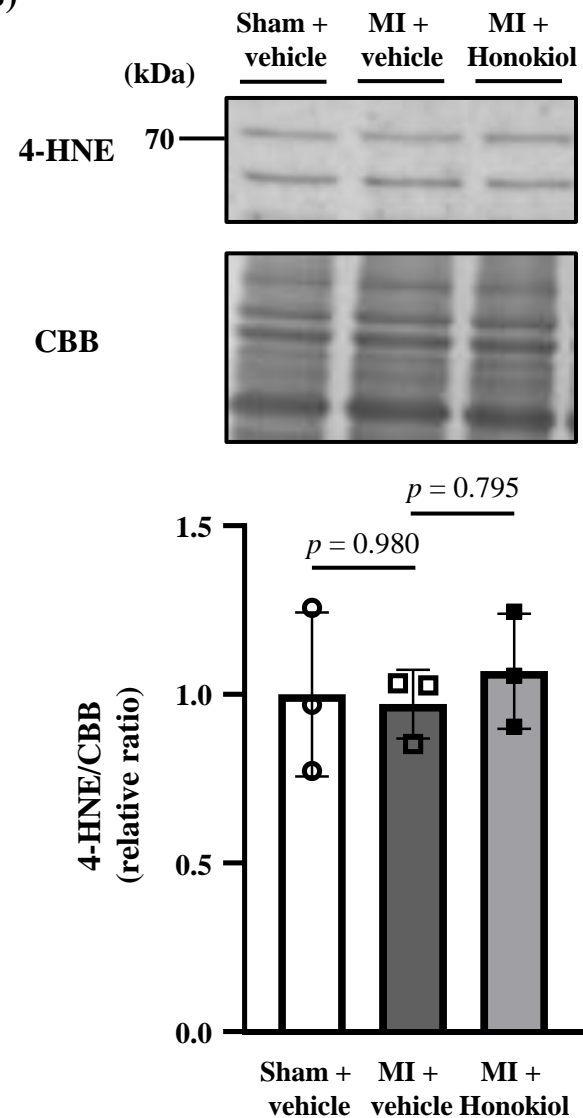

(C)

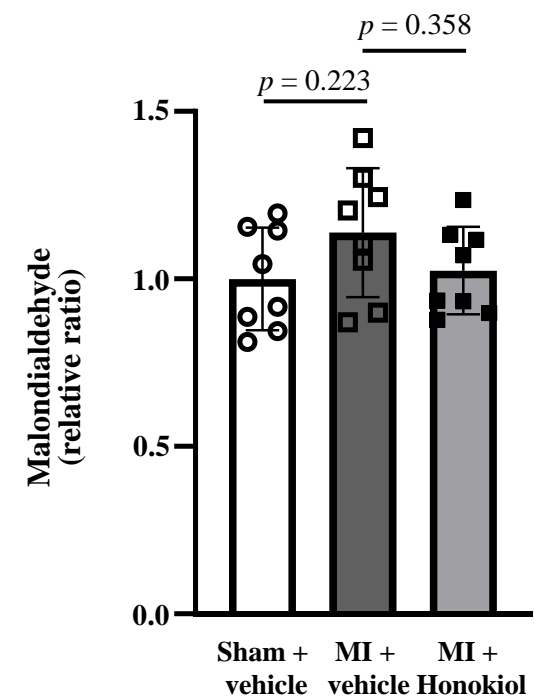

Figure S11

Supplement: Supplementary file 11 — Figure S11. Lipid peroxidation in the skeletal muscle after MI. (A) Representative western blots (top) and summary data (bottom) of acrolein in the gastrocnemius muscle of sham + vehicle (n = 7), MI + vehicle (n = 7), and MI + Honokiol mice (n = 7). (B) Representative western blots (top) and summary data (bottom) of 4‐HNE in the gastrocnemius muscle of sham + vehicle (n = 3), MI + vehicle (n = 3) and MI + Honokiol mice (n = 3). (C) Summary data of malondialdehyde in the gastrocnemius muscle of sham + vehicle (n = 8), MI + vehicle (n = 8), and MI + Honokiol mice (n = 8). Acrolein and 4‐HNE were normalized to non‐specific bands of the CBB‐stained gel. Data are shown as the mean ± SD p values were calculated by one‐way ANOVA followed by the Tukey post hoc test. MI, myocardial infarction; CBB, Coomassie Brilliant Blue; 4‐HNE, 4‐hydroxynonenal. [file JCSM-16-e13850-s010.pdf]

(A)

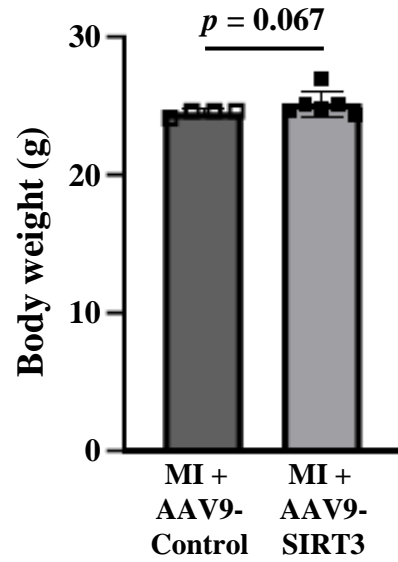

(B)

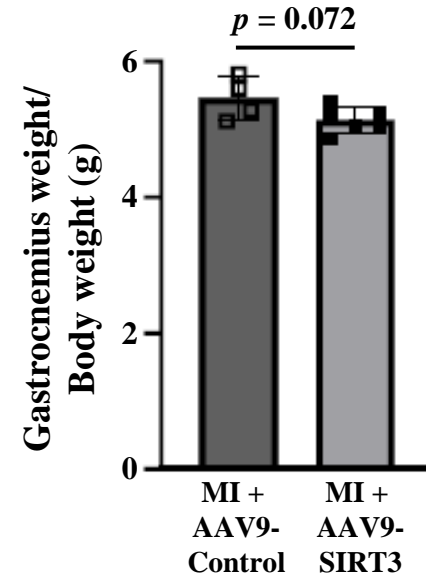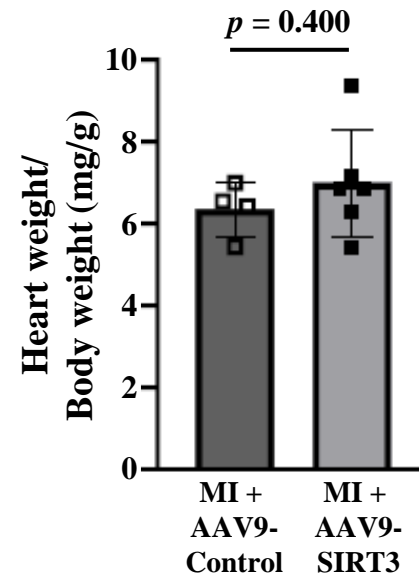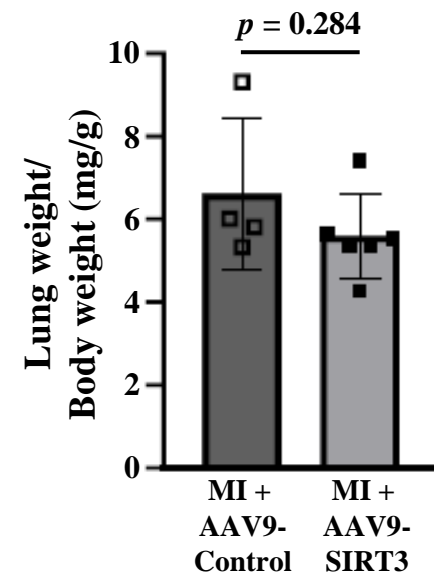

(C)

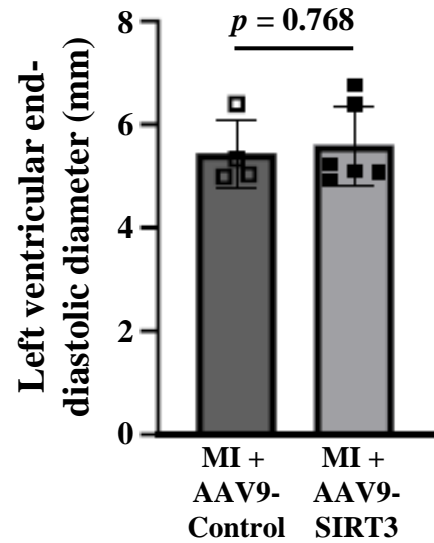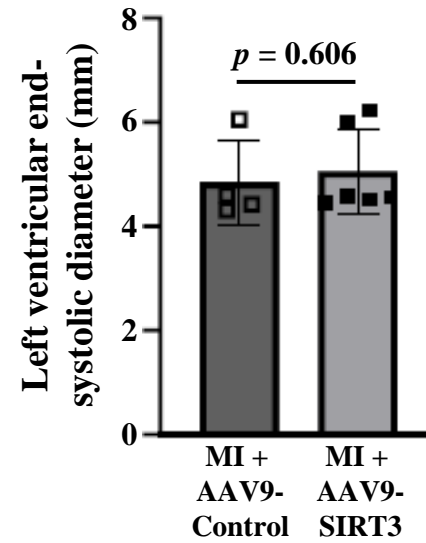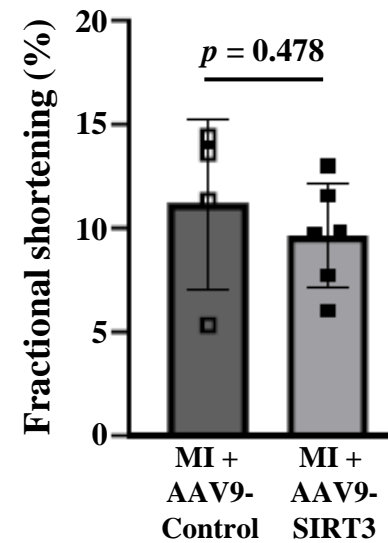

(D)

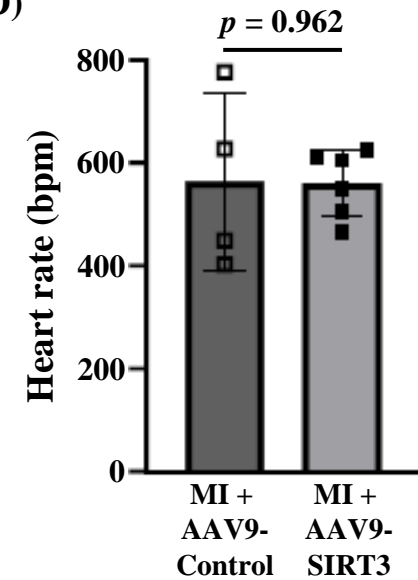

Figure S12

Supplement: Supplementary file 12 — Figure S12. Echocardiographic data and organ weight in MI mice treated with AAV9‐Control or AAV‐SIRT3. Summary data of body weight (A), and gastrocnemius weight/body weight, heart weight/body weight and lung weight/body weight (B), and left ventricular end‐diastolic diameter, left ventricular end‐systolic diameter and fractional shortening (C), and heart rate (D) in MI + AAV9‐Control (n = 4) and MI + AAV9‐SIRT3 mice (n = 6). Data are shown as the mean ± SD p values were calculated by the unpaired Student t‐test. AAV9, adeno‐associated virus serotype 9; SIRT3, sirtuin 3; MI, myocardial infarction. [file JCSM-16-e13850-s002.pdf]
