# Supplementary material for: Reduction in Acetylation of Superoxide Dismutase 2 in Skeletal Muscle Improves Exercise Capacity in Mice With Heart Failure
Source: J Cachexia Sarcopenia Muscle. 2025 Jun 13;16(3):e13850. doi: 10.1002/jcsm.13850 (PMC12163645; doi:10.1002/jcsm.13850)
Supplement: Supplementary file 4 — Figure S4. Echocardiographic data of sham and MI mice treated with vehicle or Honokiol 4 weeks after surgery Representative M‐mode echocardiographic images (A) and summary data of left ventricular end‐diastolic diameter (B), left ventricular end‐systolic diameter (C), fractional shortening (D), and heart rate (E) in sham + vehicle (n = 8), sham + Honokiol (n = 7), MI + vehicle (n = 8), and MI + Honokiol mice (n = 6). Data are shown as the mean ± SD p values of the main effect for each factor and interaction effect between two factors were calculated by two‐way ANOVA with the factors of MI and Honokiol. ANOVA with the factors of MI and Honokiol. MI, myocardial infarction. [file JCSM-16-e13850-s001.pdf]

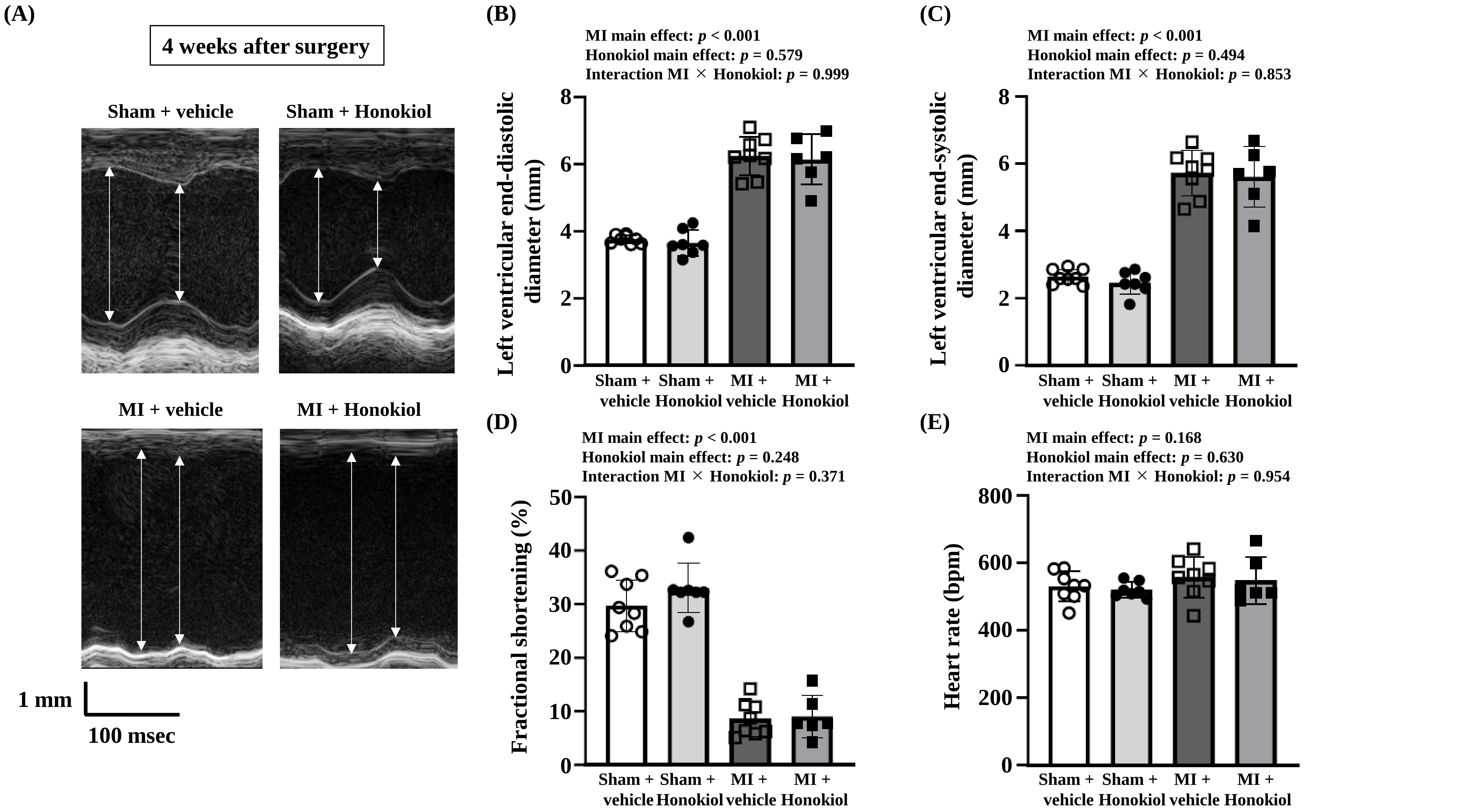
(A)
4 weeks after surgery
Sham + vehicle
Sham + Honokiol
MI + vehicle
MI + Honokiol
1 mm
100 msec
(B)
MI main effect: p < 0.001
Honokiol main effect: p = 0.579
Interaction MI × Honokiol: p = 0.999
Left ventricular end-diastolic diameter (mm)
0
2
4
6
8
Sham + vehicle
Sham + Honokiol
MI + vehicle
MI + Honokiol
(C)
MI main effect: p < 0.001
Honokiol main effect: p = 0.494
Interaction MI × Honokiol: p = 0.853
Left ventricular end-systolic diameter (mm)
0
2
4
6
8
Sham + vehicle
Sham + Honokiol
MI + vehicle
MI + Honokiol
(D)
MI main effect: p < 0.001
Honokiol main effect: p = 0.248
Interaction MI × Honokiol: p = 0.371
Fractional shortening (%)
0
10
20
30
40
50
Sham + vehicle
Sham + Honokiol
MI + vehicle
MI + Honokiol
(E)
MI main effect: p = 0.168
Honokiol main effect: p = 0.630
Interaction MI × Honokiol: p = 0.954
Heart rate (bpm)
0
200
400
600
800
Sham + vehicle
Sham + Honokiol
MI + vehicle
MI + Honokiol

Figure S4
