# Supplementary material for: Reduction in Acetylation of Superoxide Dismutase 2 in Skeletal Muscle Improves Exercise Capacity in Mice With Heart Failure
Source: J Cachexia Sarcopenia Muscle. 2025 Jun 13;16(3):e13850. doi: 10.1002/jcsm.13850 (PMC12163645; doi:10.1002/jcsm.13850)
Supplement: Supplementary file 13 — Figure S13. Treadmill system and exercise protocol The treadmill system (A) and exercise protocol (B) were shown. Running distance (meters) was measured as the distance during exercise in a direction along the treadmill. Run time (seconds) was expressed as the time from the end of warm‐up to exhaustion. Vertical distance (meters) was calculated by multiplying the running distance (meters) by sin 10°. The work (Joules) performed by the mice during exercise was calculated by vertical distance (meters) by body weight (kilograms) and then by standard gravitational acceleration (meters per second squared). g, standard gravitational acceleration. [file JCSM-16-e13850-s013.pdf]

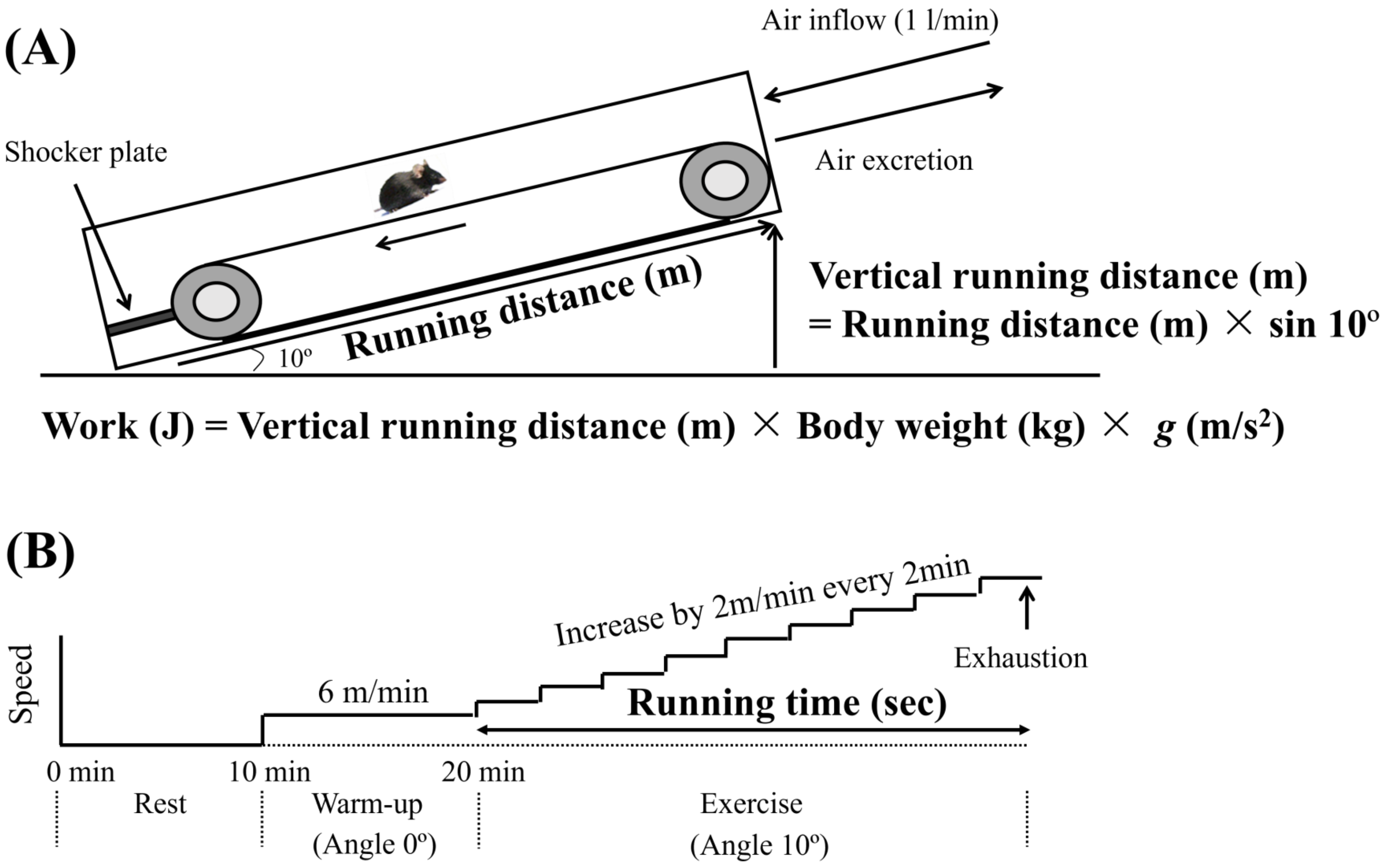
(A)
Air inflow (1 l/min)
Shocker plate
Air excretion
Running distance (m)
10º
Vertical running distance (m)
= Running distance (m) × sin 10º
Work (J) = Vertical running distance (m) × Body weight (kg) × g (m/s²)
(B)
Increase by 2m/min every 2min
Exhaustion
Speed
6 m/min
Running time (sec)
0 min
10 min
20 min
Rest
Warm-up
(Angle 0º)
Exercise
(Angle 10º)

**Figure S13**
