# Supplementary material for: Reduction in Acetylation of Superoxide Dismutase 2 in Skeletal Muscle Improves Exercise Capacity in Mice With Heart Failure
Source: J Cachexia Sarcopenia Muscle. 2025 Jun 13;16(3):e13850. doi: 10.1002/jcsm.13850 (PMC12163645; doi:10.1002/jcsm.13850)
Supplement: Supplementary file 14 — Data S1. Supplementary Information. [file JCSM-16-e13850-s009.pdf]

Supplemental methods

Echocardiography

Under light anesthesia with 1% to 2% isoflurane (Viatris, Canonsburg, PA), two-dimensional targeted M-mode images were obtained from the short axis view at the papillary muscle level using a Vevo 2100 ultrasonography system (Visual Sonics, Toronto). Left ventricular diastolic/systolic dimensions, and left ventricular fractional shortening were assessed. Fractional shortening was calculated using the following equation: % fractional shortening = [(left ventricular diastolic dimension - left ventricular systolic dimension)/left ventricular diastolic dimension] × 100

Treadmill test and lactate measurement

Mice were subjected to the treadmill test to measure indexes defining whole body exercise capacity, as previously described with a minor modification [1, 2]. The treadmill system and exercise protocol are shown in the **Supplemental Figure 13**. Namely, at the time of treadmill testing, each mouse was placed on a treadmill enclosed by a metabolic chamber through which air was flowing at a constant speed (1 l/min) (Oxymax 2; Columbus Instruments, Columbus, OH). After a 10 min warm-up at 6 m/min at 0° inclination, the angle was fixed at 10° and the speed was incrementally increased by 2 m/min until the mouse reached exhaustion. Exhaustion was defined as spending more than 10 sec on the shocker plate without attempting to re-engage in running. The work performed by the mice during exercise was calculated based on the vertical running distance using the following formula: Work (J) = Vertical running distance (m) × Body weight (kg) × g (m/s^2)

Blood lactate levels were determined using one drop of blood obtained from the tails of mice at rest, and at the end of exercise in the treadmill test [1, 2]. Lactate concentrations were assayed using a Lactate Pro-LT portable lactate blood analyzer (Arkray, Kyoto) according to the manufacturer's instruction.

Preparation of isolated mitochondria

Mitochondria were isolated from the gastrocnemius muscle, as previously described [3]. All procedures were done on ice. Gastrocnemius muscle tissues were minced and rinsed with mitochondrial isolation medium containing 100 mmol/l sucrose, 100 mmol/l KCl, 50 mmol/l Tris-HCl, 1 mmol/l KH_2PO_4, 0.1 mmol/l EGTA and 0.2% bovine serum albumin (BSA) (pH 7.4), followed by an incubation with 0.1 mg/ml protease (Nagarse) for 2 min. The muscle tissues were gently homogenized with six strokes using a Teflon pestle in a glass chamber. The homogenate was centrifuged at 800 × *g* for 10 min. The supernatant was centrifuged at 10,000 × *g* for 10 min, and the pellet was washed and centrifuged at 800 × *g* for 10 min. The supernatant was centrifuged at 7,000 × *g* for 3 min. The final pellet was suspended in suspension medium containing 225 mmol/l mannitol, 75 mmol/l sucrose, 10 mmol/l Tris, and 0.1 mmol/l EDTA (pH 7.4). Finally, the mitochondrial protein concentration was measured by the bicinchoninic acid assay.

Measurement of oxygen consumption rate

Oxygen consumption rate (OCR) was measured in the isolated mitochondria at 37°C using the Seahorse XFp analyzer (Agilent Technologies, Santa Clara, CA). After the addition of the isolated mitochondria (1 μg) to the well of a microplate, substrates and adenosine diphosphate (ADP) were added from injection ports in the following order:

(1) glutamate (200 mmol/l) + malate (40 mmol/l) (complex I-linked substrates), (2) ADP (50 mmol/l) + $MgCl_2$ (30 mmol/l), and (3) succinate (25 mmol/l) (a complex II-linked substrate). The OCR was expressed as pmol/min/μg mitochondrial protein. Data were analyzed on the Wave 2.3.0 software (Agilent Technologies).

Fiber-type staining in the gastrocnemius muscle

The myosin heavy chain (MHC) immunofluorescence staining was performed as described [4]. After excited, the gastrocnemius muscle was embedded into optimal cutting temperature compound (Sakura Finetek Japan Co., Ltd., Tokyo, Japan), and immediately frozen in isopentane and prechilled in liquid nitrogen. The frozen tissues were cut into 10 μm thick sections using a cryostat (CryoStar NX70, Thermo Fisher Scientific, Waltham, MA). Frozen sections were air-dried for 10 min, and fixed with 4% paraformaldehyde in phosphate-buffered saline (PBS) for 5 min. After washing the section with PBS (3 × 5 min), sections were incubated with 0.5% Triton in PBS for 5 min. After washing the section with PBS (3 × 5 min), the sections were pre-incubated for 60 min with 1% goat serum (Sigma Aldrich, St Louis, MO) for blocking at room temperature. The primary antibodies were used for detecting MHC isoforms; BA-F8 (specific to MHCI, type I fiber; dilution 1:50), SC-71 (specific to MHCIIA, type IIa fiber; dilution 1:600), and BF-F3 (specific to MHCIIB, type IIb fiber; dilution 1:100) (Developmental Studies Hybridoma Banks, Iowa, IA). For multicolor immunofluorescence, the secondary antibodies ware used Alexa Fluor 350 (A-21140), 488 (A-21121), and 555 (A-21426) (dilution 1:500; Thermo Fisher Scientific). Primary and secondary antibodies were applied in a cocktail to the sections, respectively. All sections were incubated with the primary antibody cocktail for 120 min at room

temperature in humidified environment. After washing the section with PBS (3 × 5 min), the secondary antibody cocktail was applied, and incubated for 60 min. After washing with PBS, the sections were visualized with a BZ-X800 fluorescence microscope (Keyence, Osaka, Japan).

Quantitative real-time PCR reaction

Total RNA extraction and quantitative polymerase chain reaction (qPCR) were performed as described previously, with some modifications [5]. Briefly, total RNA was extracted using an RNeasy Mini Kit (Qiagen, Hilden), RNA was converted to cDNA using ReverTra Ace qPCR RT Kit (TOYOBO, Osaka), and the reactions were run in an Applied Biosystems QuantStudio3 (Thermo Fisher Scientific, Waltham, MA) for the THUNDERBIRD SYBR qPCR Mix (TOYOBO). The forward (F) and reverse (R) primer sequences were as follows:

18S, F 5’-TTCTGGCCAACGGTCTAGACAAC-3’, R 5’-CCAGTGGTCTTGGTGTGCTGA-3‘;

myh1, F 5’-CGGAGTCAGGTGAATACTCACG-3’, R 5’-GAGCATGAGCTAAGGCACTCT -3’;

myh2, F 5’- ACTTTGGCACTACGGGGAAAC-3’, R 5’-CAGCAGCATTTCGATCAGCTC-3’;

myh4, F 5’- AAACCACCTCAGAGTTGTGGA-3’, R 5’-GTTCCGAAGGTTCCTGATTGC-3’; and

myh7, F 5’-ACTGTCAACACTAAGAGGGTCA-3’, R 5’-TTGGATGATTTGATCTTCCAGGG-3’

Measurement of citrate synthase activity

Frozen muscle samples were homogenized in 20 mmol/l phosphate, 0.5 mmol/l EDTA, and 5 mmol/l β-mercaptoethanol, pH 7.4. Citrate synthase activity was determined as previously described, with some modifications [1, 6]. Briefly, 30 µl of homogenate (1 mg/mL) was used for the analysis, and the reaction was initiated by 0.2 mM acetyl-CoA and 0.5 mmol/l oxaloacetate in 100 mmol/l Tris buffer and 0.1 mmol/l DTNB. Absorbance at 412 nm was followed for three min using a Varioskan LUX Multimode Microplate Reader (Thermo Fisher Scientific).

Transmission electron microscopy

Each skeletal muscle sample was fixed in 3% glutaraldehyde with 0.1 mmol/L phosphate buffer. The sample was postfixed in 2% osmium tetroxide with 0.1 mmol/L phosphate buffer, and then serially dehydrated in ethanol and embedded in epoxy resin with an automated tissue processor (EM TP; Leica, Tokyo). Sections were cut on an ultramicrotome (EM UC7; Leica, Tokyo), and the ultrathin sections were stained and observed using an electron microscope (HT7700; Hitachi, Tokyo). The number of interfibrillar mitochondria per field of μm^2 and mitochondrial cross-sectional area in gastrocnemius muscle were quantified and mitochondrial circularity was computed as width/length using the Image J program (National Institutes of Health, Bethesda, MD).

Mitochondrial hydrogen peroxide (H_2O_2) release

H_2O_2 release from isolated mitochondria was measured at 37 °C by a fluorescence spectrophotometer (Hitachi F-2500) as described previously [7]. H_2O_2 reacts with Amplex® Red reagent (Thermo Fisher Scientific) at an equal stoichiometry, catalyzed

by horseradish peroxidase (HRP), which yields the fluorescent compound resorufin (excitation: 560 nm; emission: 590 nm). After the addition of isolated mitochondria to a cuvette, Amplex red (5 μmol/l), HRP (12 U/ml), and superoxide dismutase (SOD, 45 U/ml) were added. The reaction was initiated by addition of succinate (5 mmol/l). Resorufin was monitored throughout the experiment. Before the experiment, five different concentrations of H_2O_2 were added to establish a standard curve in advance. H_2O_2 release rates from isolated mitochondria are expressed as nanomoles per minute per milligram of mitochondrial protein.

Measurement of aconitase activity

For measurement of aconitase activity, 50 mg of gastrocnemius muscle was homogenized in 200 μl PBS. Homogenates were centrifuged at 800 *g* for 10 min. Aconitase activity was measured using an assay kit (BioAssay Systems, Hayward, CA), according to manufactures' protocol.

Measurements of malondialdehyde (MDA)

MDA was measured in the gastrocnemius muscle tissues using the thiobarbituric acid reactive substances assay kit (Cayman Chemical), as previously described [8]. Briefly, thiobarbituric acid (TBA) was reacted with MDA in the samples at 100°C, and MDA-TBA adduct was fluorometrically measured (an excitation wavelength of 530 nm and an emission wavelength of 550 nm) using a Varioskan LUX Multimode Microplate Reader (Thermo Fisher Scientific).

Analysis of green fluorescent protein (GFP)

To evaluate the organ specificity of the adeno-associated virus serotype 9 (AAV9) vector, the expression of GFP in various organs including gastrocnemius muscle, heart, and liver, was observed. Each tissue was embedded into optimal cutting temperature compound (Sakura Finetek Japan Co., Ltd., Tokyo), and immediately frozen in isopentane (Fujifilm Wako Pure Chemical Corportation, Osaka) and prechilled in liquid nitrogen. The frozen tissues were cut into 10 μm thick sections using a cryostat (CryoStar NX70, Thermo Fisher Scientific), and the sections were observed using a fluorescence microscope (BZ-X800, Keyence, Osaka).

Western blotting

Western blotting was performed as described, with some modifications [1, 5, 9]. Briefly, frozen gastrocnemius muscle and cardiac muscle were homogenized with Cell Lysis Buffer (Cell Signaling Technology, Danvers, MA) supplemented with protease inhibitor cocktail (Roche, Basel), 1 mmol/l phenylmethylsulfonyl fluoride (Cell Signaling Technology), and deacetylase inhibitors (10 mmol/l nicotinamide, 1 μmol/l trichostatin A, and 5 mmol/l sodium butyrate, MedChemExpress, Monmouth Junction, NJ). After sonification and centrifugation at 13,200 *g* for 10 min at 4 °C, the supernatants were collected. Concentrations of the protein aliquots were measured using the total protein assay (Pierce BCA, Rockford, IL), and lysates (25 to 30 μg) were loaded onto 10% polyacrylamide gels, electrophoretically separated by sodium dodecyl sulfate-polyacrylamide gel electrophoresis using running buffer, and transferred by electroblotting onto a nitrocellulose membrane using transfer buffer at 100 V for 1 hour. After blocking in TBS buffer using 0.1% Tween-20 in 5% nonfat dry milk, the membranes were incubated overnight at 4°C with primary antibodies, which are listed

in the Tabel below. After washing three times in TBST buffer, the membranes were incubated with secondary antibodies, which are listed below, for 1 hour at room temperature. The membranes were washed again in TBST, and exposed by the enhanced chemiluminescence method and signal intensities were quantified by Fusion Capt software (Vilber Lourmat, Marne-la-Vallée). The quantification of protein levels was performed using the Image J program (National Institutes of Health, Bethesda, MD) and Western blotting bands were normalized using nonspecific bands stained with Coomassie Brilliant Blue (Nacalai Tesque, Kyoto), which represents the total protein amount.

**Description**	**Dilution**	**Source**	**Identifier #**
**Primary antibodies**			
Anti-SIRT4 Rabbit	1:5000	Cell Signaling Technology	69786
Anti-SIRT5 Rabbit	1:5000	Cell Signaling Technology	8782
Anti-SIRT3 Rabbit	1:5000	Cell Signaling Technology	5490
Anti-acetylated lysine Rabbit	1:3000	Cell Signaling Technology	9441
Anti-SOD2 Rabbit	1:3000	Abcam	13533
Anti-acetylated SOD2 (68) Rabbit	1:3000	Abcam	137037
Anti-PGC-1α Mouse	1:3000	Abcam	191838
Anti-SIRT1 Rabbit	1:5000	Cell Signaling Technology	9475
Anti-Mitofusin1 Mouse	1:10000	Abcam	57602
Anti-Mitofusin2 Mouse	1:10000	Abcam	56889
Anti-optic atrophy1 Mouse	1:5000	BD Biosciences	612606
Anti-DRP1 Mouse	1:5000	BD Biosciences	611112
Anti-PINK1 Mouse	1:5000	Abcam	75487
Anti- Parkin Mouse	1:5000	Abcam	77924
GCN5L1 Rabbit	1:3000	Proteintech	19687-1-AP

Anti-acrolein Mouse	1:1000	Japan Institution for the Control of Aging	MAR-020n
Anti-4-HNE Rabbit	1:3000	Abcam	46545
Anti-GFP Rabbit	1:3000	Cell Signaling Technology	2956
**Secondary antibodies**			
Anti-rabbit IgG conjugated with horseradish peroxidase	1:5000	Cell Signaling Technology	7074
Anti-mouse IgG conjugated with horseradish peroxidase	1:5000	Cell Signaling Technology	7076

SIRT, sirtuins; SOD2, superoxide dismutase 2; PGC-1α, peroxisome proliferator-activated receptor γ coactivator-1 α; DRP1, dynamin-related protein 1; PINK1, PTEN-induced serine/threonine kinase1; GCN5L1, general control of amino acid synthesis 5 like 1; 4-HNE, 4-hydoxynenenal; GFP, green fluorescent protein

Supplemental references

1. Tsuda M, Fukushima A, Matsumoto J, Takada S, Kakutani N, Nambu H, et al. Protein acetylation in skeletal muscle mitochondria is involved in impaired fatty acid oxidation and exercise intolerance in heart failure. *J Cachexia Sarcopenia Muscle.* 2018;**9**(5):844-59.

2. Matsumoto J, Takada S, Furihata T, Nambu H, Kakutani N, Maekawa S, et al. Brain-derived neurotrophic factor improves impaired fatty acid oxidation via the activation of adenosine monophosphate-activated protein kinase-α - proliferator-activated receptor-γ coactivator-1α signaling in skeletal muscle of mice with heart failure. *Circ Heart Fail.* 2021;**14**(1):e005890.

3.Christiansen LB, Dela F, Koch J, Hansen CN, Leifsson PS, Yokota T. Impaired cardiac mitochondrial oxidative phosphorylation and enhanced mitochondrial oxidative stress in feline hypertrophic cardiomyopathy. *Am J Physiol Heart Circ Physiol.* 2015;**308**(10):H1237-47.

4.Yamanashi K, Kinugawa S, Fukushima A, Kakutani N, Takada S, Obata Y, et al. Branched-chain amino acid supplementation ameliorates angiotensin II-induced skeletal muscle atrophy. *Life Sci.* 2020;**250**:117593.

5. Ishikita A, Matsushima S, Ikeda S, Okabe K, Nishimura R, Tadokoro T, et al. GFAT2 mediates cardiac hypertrophy through HBP-O-GlcNAcylation-Akt pathway. *iScience.* 2021;**24**(12):103517.

6. Suwa M, Nakano H, Kumagai S. Effects of chronic AICAR treatment on fiber composition, enzyme activity, UCP3, and PGC-1 in rat muscles. *J Appl Physiol* (1985). 2003;**95**(3):960-8.

7.Sahlin K, Shabalina IG, Mattsson CM, Bakkman L, Fernström M, Rozhdestvenskaya Z, et al. Ultraendurance exercise increases the production of reactive oxygen species in isolated mitochondria from human skeletal muscle. *J Appl Physiol* (1985). 2010;**108**(4):780-7.

8.Tadokoro T, Ikeda M, Ide T, Deguchi H, Ikeda S, Okabe K, et al. Mitochondria-dependent ferroptosis plays a pivotal role in doxorubicin cardiotoxicity. *JCI Insight.* 2020;**5**(9):e132747.

9. Takada S, Masaki Y, Kinugawa S, Matsumoto J, Furihata T, Mizushima W, et al. Dipeptidyl peptidase-4 inhibitor improved exercise capacity and mitochondrial biogenesis in mice with heart failure via activation of glucagon-like peptide-1 receptor signalling. *Cardiovasc Res.* 2016;**111**(4):338-47.

Supplemental Figure 1. SIRT4 and SIRT5 expressions in the skeletal muscle

Representative western blots and summary data of SIRT4 and SIRT 5 in the gastrocnemius muscle of sham + vehicle (n = 6) and MI + vehicle (n = 6). The blots were normalized to the nonspecific bands of CBB-stained gel. Data are shown as the mean ± SD. *p*-values were calculated by the unpaired Student *t*-test. MI, myocardial infarction; CBB, SIRT4, sirtuin 4; SIRT5, sirtuin 5; Coomassie Brilliant Blue.

Supplemental Figure 2. Acetylated lysine in the mitochondrial lysates

(A) Representative western blot (left) and summary data (right) of acetylated lysine in the mitochondrial lysates from C2C12 myotubes treated with vehicle (n = 7) and Honokiol (n = 7). (B) Representative western blot (left) and summary data (right) of acetylated lysine in the mitochondrial lysates from C2C12 myotubes treated with vehicle (n = 4) and Honokiol (n = 4) with transfection of SIRT3 siRNA. Results were normalized to non-specific bands of the CBB-stained gel. Data are shown as the mean ± SD. *p*-values were calculated by the unpaired Student *t*-test. CBB, Coomassie Brilliant Blue

Supplemental Figure 3. Echocardiographic data of sham and MI mice before treatment with vehicle or Honokiol 2 weeks after surgery

Summary data of left ventricular end-diastolic diameter (A), left ventricular end-systolic diameter (B), fractional shortening (C), and heart rate (D) in sham + vehicle (n = 8), sham + Honokiol (n = 7), MI + vehicle (n = 8), and MI + Honokiol mice (n = 6). Data are shown as the mean ± SD. *p*-values of the main effect for each factor and interaction

effect between two factors were calculated by two-way ANOVA with the factors of MI and Honokiol. MI, myocardial infarction

Supplemental Figure 4. Echocardiographic data of sham and MI mice treated with vehicle or Honokiol 4 weeks after surgery

Representative M-mode echocardiographic images (A) and summary data of left ventricular end-diastolic diameter (B), left ventricular end-systolic diameter (C), fractional shortening (D), and heart rate (E) in sham + vehicle (n = 8), sham + Honokiol (n = 7), MI + vehicle (n = 8), and MI + Honokiol mice (n = 6). Data are shown as the mean ± SD. *p*-values of the main effect for each factor and interaction effect between two factors were calculated by two-way ANOVA with the factors of MI and Honokiol. MI, myocardial infarction

Supplemental Figure 5. Organ weights in MI mice and sham mice treated with vehicle or Honokiol

Summary data of heart weight/body weight (A), and lung weight/body weight (B) in sham + vehicle (n = 8), sham + Honokiol (n = 7), MI + vehicle (n = 8), and MI + Honokiol mice (n=6). Data are shown as the mean ± SD. *p*-values of the main effect for each factor, and interaction effect between two factors were calculated by two-way ANOVA with the factors of MI and Honokiol. MI, myocardial infarction

Supplemental Figure 6. Immunofluorescence staining and gene expression of MHC

(A) Summary data of the proportion of type IIa (top) and type IIb (bottom) fibers in the gastrocnemius muscle of sham + vehicle (n = 5), MI + vehicle (n = 5), and MI +

Honokiol (n = 5) to the total fibers. (B) Summary data of gene expression of *Myh7*, *Myh2*, *Myh1*, and *Myh4* in the gastrocnemius muscle of sham + vehicle (n = 8), MI + vehicle (n = 7), and MI + Honokiol mice (n = 8). The expression of each gene was normalized to that of *18S*. Data are shown as the mean ± SD. *p*-values were calculated by one-way ANOVA followed by the Tukey *post hoc* test. MI, myocardial infarction

Supplemental Figure 7. Expression of proteins associated with mitochondrial biogenesis, fission, fusion, and mitophagy

Representative western blots (A) and summary data (B) of PGC-1α, SIRT1, Mitofusin1, Mitofusin2, Optic atrophy1, DRP1, PINK1, and Parkin in the gastrocnemius muscle of sham + vehicle (n = 6), MI + vehicle (n = 6), and MI + Honokiol (n = 6). The blots were normalized to the nonspecific bands of CBB-stained gel. Data are shown as the mean ± SD. *p*-values were calculated by one-way ANOVA followed by the Tukey *post hoc* test. MI, myocardial infarction; PGC-1α, peroxisome proliferator-activated receptor γ coactivator-1 α; SIRT1, sirtuin 3; DRP1, dynamin related protein 1; PINK1, PTEN-induced serine/threonine kinase1; CBB, Coomassie Brilliant Blue.

Supplemental Figure 8. Acetylated lysine in the mitochondrial lysates

Representative western blot (left) and summary data (right) of acetylated lysine in the mitochondrial lysates from the skeletal muscle of sham + vehicle (n = 7), MI + vehicle (n = 8), and MI + Honokiol mice (n = 8). Results were normalized to non-specific bands of the CBB-stained gel. Data are shown as the mean ± SD. *p*-values were calculated by one-way ANOVA followed by the Tukey *post hoc* test. MI, myocardial infarction; CBB, Coomassie Brilliant Blue

Supplemental Figure 9. GCN5L1 expression in sham mice and MI mice

Representative western blots and summary data of GCN5L1 in the gastrocnemius muscle of sham + vehicle (n = 5) and MI + vehicle (n = 4). Results were normalized to the nonspecific bands of CBB-stained gel. Data are shown as the mean ± SD. *p*-values were calculated by the unpaired Student *t*-test. MI, myocardial infarction; GCN5L1, general control of amino acid synthesis 5 like 1; CBB, Coomassie Brilliant Blue.

Supplemental Figure 10. SIRT3 expression and acetylated SOD2 in the heart

Representative western blots (left) and summary data (right) of SIRT3 and acetylated SOD2 in the Heart of sham + vehicle (n = 4), MI + vehicle (n = 4), and MI + Honokiol mice (n = 4). SIRT3 was normalized to non-specific bands of the CBB-stained gel. Acetylated SOD2 was normalized to total SOD2. Data are shown as the mean ± SD. *p*-values were calculated by one-way ANOVA followed by the Tukey *post hoc* test. MI, myocardial infarction; SIRT3, sirtuin 3, SOD2, superoxide dismutase 2; CBB, Coomassie Brilliant Blue.

Supplemental Figure 11. Lipid peroxidation in the skeletal muscle after MI

(A) Representative western blots (top) and summary data (bottom) of acrolein in the gastrocnemius muscle of sham + vehicle (n = 7), MI + vehicle (n = 7), and MI + Honokiol mice (n = 7). (B) Representative western blots (top) and summary data (bottom) of 4-HNE in the gastrocnemius muscle of sham + vehicle (n = 3), MI + vehicle (n = 3), and MI + Honokiol mice (n = 3). (C) Summary data of malondialdehyde in the gastrocnemius muscle of sham + vehicle (n = 8), MI + vehicle (n = 8), and MI +

Honokiol mice (n = 8). Acrolein and 4-HNE were normalized to non-specific bands of the CBB-stained gel. Data are shown as the mean ± SD. *p*-values were calculated by one-way ANOVA followed by the Tukey *post hoc* test. MI, myocardial infarction; CBB, Coomassie Brilliant Blue; 4-HNE, 4-hydroxynonenal

Supplemental Figure 12. Echocardiographic data and organ weight in MI mice treated with AAV9-Control or AAV-SIRT3

Summary data of body weight (A), and gastrocnemius weight/body weight, heart weight/body weight, and lung weight/body weight (B), and left ventricular end-diastolic diameter, left ventricular end-systolic diameter, and fractional shortening (C), and heart rate (D) in MI + AAV9-Control (n = 4) and MI + AAV9-SIRT3 mice (n = 6). Data are shown as the mean ± SD. *p*-values were calculated by the unpaired Student *t*-test. AAV9, adeno-associated virus serotype 9; SIRT3, sirtuin 3; MI, myocardial infarction.

Supplemental Figure 13. Treadmill system and exercise protocol

The treadmill system (A) and exercise protocol (B) were shown. Running distance (m) was measured as the distance during exercise in a direction along the treadmill. Run time (s) was expressed as the time from the end of warm-up to exhaustion. Vertical distance (m) was calculated by multiplying the running distance (m) by sin 10°. The work (J) performed by the mice during exercise was calculated by vertical distance (m) by body weight (kg) and then by standard gravitational acceleration (m/s^2). *g*, standard gravitational acceleration.
